# Emerging Thermal Detectors Based on Low-Dimensional Materials: Strategies and Progress

**DOI:** 10.3390/nano15060459

**Published:** 2025-03-18

**Authors:** Yang Peng, Jun Liu, Jintao Fu, Ying Luo, Xiangrui Zhao, Xingzhan Wei

**Affiliations:** 1 Chongqing Institute of Green and Intelligent Technology, Chinese Academy of Sciences, Chongqing 400714, China; 2 Chongqing School, University of Chinese Academy of Sciences, Chongqing 400714, China; 3 School of Optoelectronic Science and Engineering, University of Electronic Science and Technology of China, Chengdu 610054, China; 4 Hangzhou Hikmicro Sensing Technology Co., Ltd., Hangzhou 311599, China; 5 School of Optoelectronic Engineering, Chongqing University of Posts and Telecommunications, Chongqing 400065, China; 6 University of Chinese Academy of Sciences, Beijing 100049, China

**Keywords:** thermal detector, low-dimensional material, metasurface, optical communication, multidimensional photodetection

## Abstract

Thermal detectors, owing to their broadband spectral response and ambient operating temperature capabilities, represent a key technological avenue for surpassing the inherent limitations of traditional photon detectors. A fundamental trade-off exists between the thermal properties and the response performance of conventional thermosensitive materials (e.g., vanadium oxide and amorphous silicon), significantly hindering the simultaneous enhancement of device sensitivity and response speed. Recently, low-dimensional materials, with their atomically thin thickness leading to ultralow thermal capacitance and tunable thermoelectric properties, have emerged as a promising perspective for addressing these bottlenecks. Integrating low-dimensional materials with metasurfaces enables the utilization of subwavelength periodic configurations and localized electromagnetic field enhancements. This not only overcomes the limitation of low light absorption efficiency in thermal detectors based on low-dimensional materials (TDLMs) but also imparts full Stokes polarization detection capability, thus offering a paradigm shift towards multidimensional light field sensing. This review systematically elucidates the working principle and device architecture of TDLMs. Subsequently, it reviews recent research advancements in this field, delving into the unique advantages of metasurface design in terms of light localization and interfacial heat transfer optimization. Furthermore, it summarizes the cutting-edge applications of TDLMs in wideband communication, flexible sensing, and multidimensional photodetection. Finally, it analyzes the major challenges confronting TDLMs and provides an outlook on their future development prospects.

## 1. Introduction

Photodetectors serve as the core components for optical information sensing, providing indispensable functions in applications such as optical communication [1,2,3,4,5,6,7,8], environmental monitoring [9,10,11,12,13,14,15,16], and bioimaging [12,17,18,19,20,21,22]. Based on their operating principles, photodetectors comprise two primary types: photon (photovoltaic/photoconductive) [23,24,25,26,27,28,29,30,31,32,33] and thermal (pyroelectric/thermocouple/bolometer) [34,35,36,37,38,39,40,41,42,43,44,45,46,47,48]. Photon detectors, operating on photoelectric conversion principles, exhibit high sensitivity and rapid response speeds [49,50,51,52,53,54,55,56]. Nevertheless, the detection wavelength range of photon detectors is typically limited by the bandgap of the photosensitive materials [57,58,59,60,61,62]. The detection of mid-wave and long-wave infrared, as well as terahertz wavelengths, relies on narrow bandgap semiconductors, which are susceptible to thermal excitation noise [50,63]. Consequently, the implementation of cryogenic cooling modules within infrared photodetection systems is required [64,65,66], thereby severely constraining device integration and application scenarios. Conversely, thermal detectors utilize photothermal-thermoelectric conversion principles. The response characteristics of thermal detectors transcend the physical constraints of semiconductor bandgaps, exhibiting distinctive advantages such as a wide spectral response and room-temperature operation [67,68,69,70,71,72]. However, traditional thermal detectors suffer from inherent limitations stemming from thermal relaxation effects. Such effects give rise to sluggish response speeds (on the order of milliseconds) and diminished sensitivity.

Low-dimensional materials, encompassing two-dimensional materials [73,74,75,76,77,78], one-dimensional nanowires [79,80,81,82,83,84], and zero-dimensional quantum dots [85,86,87,88,89,90], offer potential solutions to circumvent the performance limitations of conventional thermal detectors. The quantum confinement effects and interfacial engineering characteristics of low-dimensional materials provide unique advantages [91,92,93,94,95,96,97,98,99]. Specifically, low-dimensional materials can optimize carrier relaxation pathways via quantum confinement effects. Through quantum confinement effects, low-dimensional materials can optimize the carrier relaxation pathways, suppressing phonon scattering and diminishing thermal conductivity [100]. This phenomenon generates favorable temperature gradients, which are conducive to high-sensitivity detection. Two-dimensional materials, such as graphene, exhibit ultra-low electronic heat capacity [99,101,102] and weak heat dissipation [103,104,105]. After absorbing light, their electron temperature is significantly higher than the lattice temperature, while maintaining a fast-thermal relaxation time [106,107]. Additionally, the weak van der Waals interlayer interactions in low-dimensional materials facilitate layer-by-layer stacking, enabling versatile functional modulation [108,109,110,111,112,113,114,115,116,117]. Notably, the electronic band structure of stacked materials is significantly influenced by both the number of layers and stacking configuration, including stacking order (e.g., A–B–C or A–B–A stacking) [118,119,120] and twist angles [121,122]. Through meticulously interfacial engineering via layer rotation [123], the thermal conductivity of low-dimensional materials can be reduced to levels comparable to that of air, which provides a physical basis for constructing high-sensitivity thermal detectors requiring high-temperature gradients.

Due to the unique advantages of low-dimensional materials, thermal detectors based on low-dimensional materials (TDLMs) exhibit multiple properties, including self-powering, high sensitivity, and rapid response. These advantageous properties are typically not found concurrently in conventional thermal detectors. In optical communication, TDLMs significantly enhance the response sensitivity and transmission bandwidth of terahertz to visible light communication links through precise regulation of the localized optical field distribution and photothermal conversion process [124,125]. In the field of spectral imaging, TDLMs enable simultaneous optimization of both wavelength resolution and sensitivity. Through a reconfigurable metasurface array and in conjunction with external stimuli such as electric field modulation [126,127,128,129,130], optical pumping excitation [131,132], or temperature modulation [133], dynamic control over the working wavelength and sensitivity of TDLMs can be achieved. Notably, the intrinsic optical anisotropy of two-dimensional materials, exemplified by black phosphorus, enables the synchronous analysis of spectral and polarization information within a single device [134,135]. This multi-physical quantity fusion paradigm transcends the volumetric constraints of conventional spectrometers. The remarkable mechanical properties inherent in low-dimensional materials provide a foundation for the flexible applications of TDLMs. Graphene, for instance, exhibits Young’s modulus reaching 1 TPa, enabling devices to maintain stable conductivity and structural integrity after enduring thousands of bending cycles [136]. This mechanical robustness, in conjunction with solution-based fabrication techniques, facilitates the direct printing of TDLMs onto biocompatible substrates, thereby advancing the field of wearable optoelectronics.

**Figure 1:**
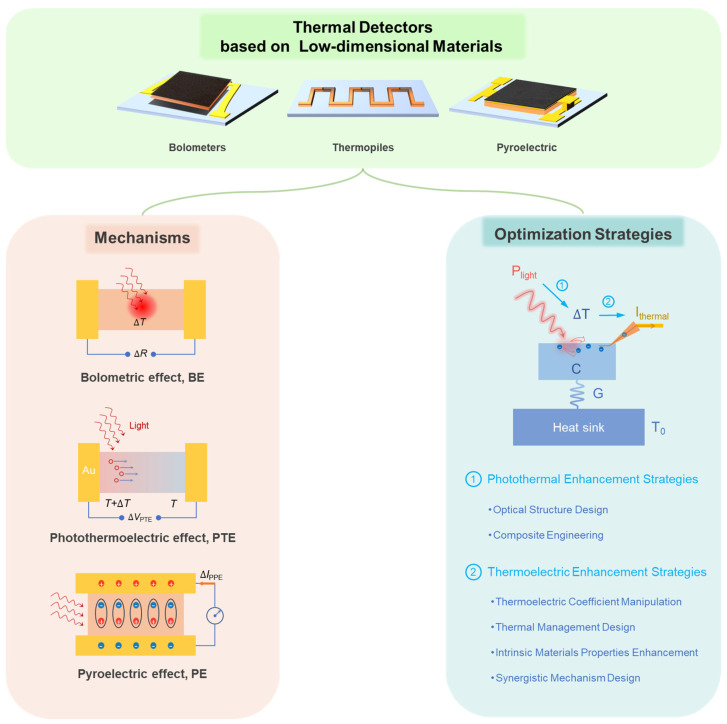
Structure, mechanism, and optimization strategies overview of TDLMs.

Herein, a review of recent advancements in TDLMs is conducted, as illustrated in Figure 1. Initially, the fundamental working principles of thermal detectors are elucidated, alongside an introduction of key performance metrics. Subsequently, the latest research breakthroughs in enhancing the performance of TDLMs are summarized, delving into optimization strategies for the underlying photothermal and thermoelectric processes. Significant progress in diverse application domains, including optical communication, multidimensional spectral imaging, and flexible sensing, is comprehensively analyzed. Finally, future development trends for TDLMs are discussed, along with a prospective analysis of the challenges that may impede future progress.

## 2. Principles and Parameters for Thermal Detector

### 2.1. Principles of Thermal Detector

The fundamental principle of the photothermoelectric (PTE) effect can be deconstructed into two core physical processes: photothermal conversion and thermoelectric conversion. Upon absorption of photons, the material generates a non-equilibrium charge carrier distribution, resulting in a localized electron temperature gradient, Δ*T_e_*, which drives directional diffusion of charge carriers from the hot to the cold end, establishing a potential difference, Δ*V_PTE_*. The phenomenon is known as the Seebeck effect. The Seebeck coefficient, defined as the ratio of Δ*V_PTE_* to Δ*t*, exhibits a close correlation with the electrical conductivity of the materials, *σ*, which can be expressed by the Mott relation [137]:

(1) S=−π2kB2T3edlnσdE E=Ef

where *k_B_* represents the Boltzmann constant, *T* denotes the absolute temperature, *e* signifies the elementary charge, and *E_f_* corresponds to the Fermi level. As carrier diffusion is driven by the spontaneous diffusion engendered by the temperature gradient, external biasing is not required for PTE detectors, thereby mitigating the influence of 1/f noise. The primary noise source in PTE detectors is Johnson–Nyquist noise, whose spectral density can be approximated as the square root of 4*Rk_B_T*, where *R* represents the electrical resistance of the device.

The bolometric (BE) effect is based on the change in resistance (Δ*R*) caused by increased material temperature due to light exposure. A fundamental distinction between the BE and PTE effects resides in the nature of the photocurrent generated. The PTE effect produces a self-driven photocurrent, analogous to its manifestation in photovoltaic devices. In contrast, the BE effect does not generate a detectable photocurrent in the absence of an external bias. While the PTE effect relies on Seebeck voltage across a heterogeneous junction, the BE effect uniquely enables full spectral response in homogeneous materials.

The pyroelectric (PE) effect is a physical phenomenon wherein spontaneous polarization in a material fluctuates in response to temperature variations. Illumination of a pyroelectric material with modulated light induces a photothermal field perturbation that modifies the vibrational energy levels within the crystal lattice, resulting in periodic variations in the spontaneous polarization intensity. The redistribution of surface-free charges within a closed circuit gives rise to a transient pyroelectric current *I_p_* = *p·A*(*dT*/*dt*), where *p* denotes the pyroelectric coefficient, and *A* represents the effective area. Comparable to the PTE effect, the PE effect enables photoelectric signal conversion without needing an external bias voltage, exhibiting inherent self-powered characteristics. In device applications, PE detectors, leveraging a dynamic thermoelectric conversion mechanism, have found widespread use in infrared spectroscopy, radiometer, and non-contact temperature sensing. However, the response mechanism of PE detectors is inherently constrained by the derivative of the temperature change rate. When the light signal reaches a steady state (*dT*/*dt*→0), the device loses its charge separation driving force, significantly reducing sensitivity to static light intensity. The intrinsic characteristic renders PE detectors more suitable for pulsed laser detection, dynamic thermography, and other applications demanding rapid response.

### 2.2. Parameters of Thermal Detector

The performance characteristics of a thermal detector directly reflect its detection capabilities. Fundamental parameters for evaluating thermal detectors include responsivity, response time, external quantum efficiency, etc. Furthermore, the figure-of-metric for thermal detectors operational status is illustrated to better explore its detection efficiency.

#### 2.2.1. Responsivity

The responsivity is defined as the ratio of photocurrent or photovoltage to incident optical power. It signifies the efficiency with which a photodetector responds to optical signals, as depicted in Formula (2) [138,139]:

(2) $$R_{I}=\frac{I_{ph}}{P_{in}\cdot A_{d}};\;R_{V}=\frac{V_{ph}}{P_{in}\cdot A_{d}}$$

where *R_I_* is the current responsivity, *R_V_* is the voltage responsivity, *I_ph_* is the photocurrent (or *V_ph_* for photovoltage), *P_in_* is the incident optical power density, and *A_d_* is the photosensitive area of the photodetector. The responsivity is widely employed to evaluate the capability of a photodetector to generate photocurrent or photovoltage in response to incident light with a specific power density and wavelength.

#### 2.2.2. Response Speed at 3 dB Working Bandwidth

The capacity of a photodetector to perceive high-frequency light signals depends on its response speed. The response speed is typically characterized by the rise time (*t_r_*) and fall time (*t_f_*), which are defined as the maximum time intervals required for the photocurrent or photovoltage to transition from 10% (90%) to 90% (10%) of full amplitudes. For photodetectors, responsivity is related to the on–off frequency of light. As the on–off frequency increases, the response speed gradually falls behind the switching speed of light, resulting in a continuous decrease in photocurrent. When the photocurrent value drops to 70.7% of its maximum, the corresponding modulation frequency represents the 3 dB working bandwidth of the photodetector.

#### 2.2.3. External Quantum Efficiency

The external quantum efficiency (*EQE*) quantifies the number of photoelectrons generated per incident photon, expressed as a percentage. *EQE* is defined as follows [138,139]:

(3) $$EQE=\frac{N_{carrier}}{N_{photon}}=\frac{I_{ph}/q}{{(P}_{in}{\cdot A}_{d})/hv}=R_{\lambda }\frac{hc}{e\lambda }$$

where *N_carrier_* is the number of carriers in the photocurrent, *N_photon_* is the number of incident photons, *q* is the elementary charge, *h* is Planck’s constant, *v* is the frequency of light, *c* is the speed of light, *λ* is the light wavelength, and *R_λ_* denotes the spectrally dependent responsivity of the photodetector. This parameter is crucial as it directly influences the photoelectric conversion efficiency of the photodetector. The *EQE* is influenced not only by the inherent material properties but also by device architecture, interfacial characteristics, and fabrication processes.

#### 2.2.4. Noise Equivalent Power

Noise, manifest as macroscopic constant current or voltage exhibiting microscopic, complex random fluctuations when amplified, is an important metric for assessing the quality of commercially viable photodetectors. Minimizing noise is paramount as it dictates the minimum detectable signal. Noise encompasses various types, including thermal noise, shot noise, and flicker noise, collectively contributing to the total noise. Noise equivalent power (*NEP*), a relevant figure of merit, is defined as the optical power required to achieve a signal-to-noise ratio of one within a specified bandwidth, expressed in W·Hz^−1/2^. *NEP* also represents the minimum detectable power and is calculated using the following formula [138,139]:

(4) $$NEP=\frac{i_{n}}{R_{I}}$$

where *i_n_* denotes the noise current.

#### 2.2.5. Specific Detectivity

The specific detectivity (D*), a critical parameter for assessing the weak light detection capabilities of photodetectors, integrates several performance metrics, including sensitivity, active area, noise characteristics, and operational bandwidth. Its widespread application in photodetector performance comparisons stems from its comprehensive nature. The calculation of D* typically employs the following formula [138,139]:

(5) $$D^{*}=\frac{\sqrt{A_{d}\cdot \Delta f}}{NEP}=R_{I}\cdot \frac{\sqrt{A_{d}\cdot \Delta f}}{i_{n}}$$

where *A_d_* and Δ*f* represent the active area and bandwidth of the photodetector, respectively. In some cases, when the noise is mainly shot noise, (2*qI_dark_*)^1/2^ is usually used to calculate the theoretical noise current (*i_n_*) of the photodetector, where *I_dark_* is the dark current of the detector.

#### 2.2.6. Figure-of-Merit for Thermal Detectors

Due to the presence of both photothermal conversion and thermoelectric conversion processes in thermal detectors, defining a comprehensive figure of merit based on intrinsic material properties is crucial for assessing device detection efficiency. Herein, material merit factors are delineated for three prevalent thermal detection principles. The performance of detectors based on the PTE effect is intrinsically linked to the inherent thermoelectric conversion efficiency of the material. Consequently, the dimensionless figure of merit (*ZT*) is defined, which is expressed as [140]

(6) $$ZT=\frac{S^{2}\sigma T}{k}$$

where *S* is the Seebeck coefficient of the material, *σ* is the electrical conductivity, *T* is the absolute temperature, and *k* is the thermal conductivity. The physical significance of the *ZT* metric lies in its ability to balance the thermoelectric output power of the material against its thermal energy dissipation. A larger Seebeck coefficient augments the open-circuit voltage output, while high electrical conductivity mitigates ohmic losses. Conversely, low thermal conductivity enhances the thermoelectric potential by establishing a large temperature gradient.

For PE detectors, the efficiency in detecting external temperature variations is gauged by the following merit factor:

(7) $$F_{d}=\frac{p}{c_{p}{\left(\varepsilon_{r}tan\delta \right)}^{1/2}}$$

where *p* is the pyroelectric coefficient of the material, *c_p_* is its volumetric specific heat capacity, *ε_r_* is its relative permittivity, and *tanδ* is its dielectric loss tangent. Given that the volumetric specific heat capacity (*c_p_*) exhibits minimal variation with composition, manipulating the remaining three parameters, *p*, *ε_r_*, and *tanδ* through compositional tailoring and ionic doping, can effectively enhance the detection efficiency.

Based on the operating principle of the BE effect, the core parameter governing the sensitivity of a bolometer is the temperature coefficient of resistance (TCR) [141]:

(8) $$TCR\left(R_{0}\right)=\frac{1}{R_{0}}\frac{dR}{dT}=-\frac{1}{I_{0}}\frac{dI}{dT}$$

where *TCR* denotes the percentage change in resistance per Kelvin at the operating point *R*_0_, which corresponds to the normalized current change per Kelvin around the operating current *I*_0_ (Equation (8)). The *TCR* for metallic bolometers is 0.4% K^−1^, while for semiconductor bolometers, it ranges from 2 to 4% K^−1^ [142].

## 3. Research Progress on TDLMs

### 3.1. Photothermal Enhancement Strategies

The photothermal process is pivotal in thermal detection, directly influencing the quantum efficiency and sensitivity of TDLM to optical signals. This process is contingent upon not only the light absorption properties of the material but also the thermal capacitance of TDLMs. Given the critical role of photothermal conversion efficiency in elevating TDLMs performance, three distinct strategies for photothermal enhancement are presented in this review.

#### 3.1.1. Optical Structure Design

**Figure 2:**
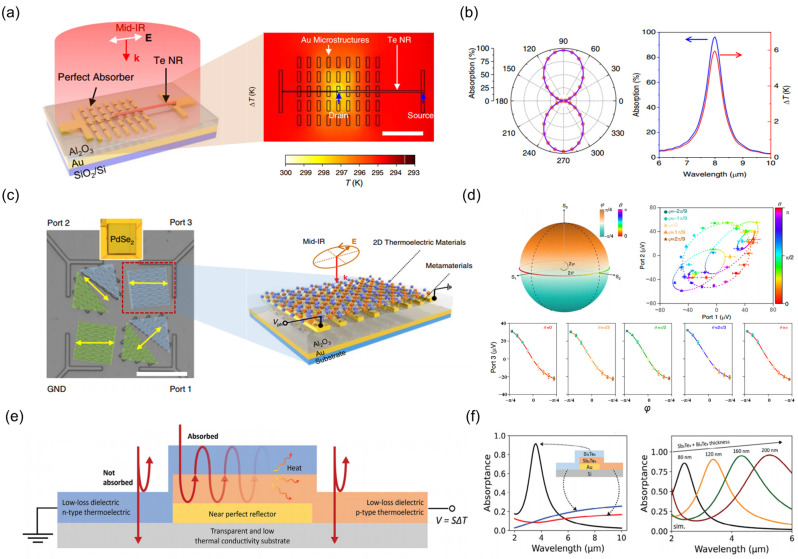
Plasmonic resonances in TDLMs. (**a**) Schematic illustration of a Te nanowire photodetector with Au plasmonic absorber. The inset depicts the simulated temperature distribution on the surface of the device. (**b**) Linear polarization angle-dependent absorbance of the device and absorption spectra/surface temperature variations at different wavelengths. (**a**,**b**) Reproduced with permission [124]. Copyright 2023, Springer Nature (Berlin, Germany). (**c**) Optical image of a device fabricated from two-dimensional PdSe_2_ material. The local inset displays a schematic of the structure where a metallic plasmonic resonator couples with a two-dimensional thermoelectric material. (**d**) Three-terminal polarization response: voltage variation as a function of azimuthal angle (*θ*) and ellipticity angle (*φ*) for full Stokes polarization detection. (**c**,**d**) Reproduced with permission [143]. Copyright 2022, Springer Nature. (**e**) Schematic illustration of a TDLM based on Sb_2_Te_3_-Bi_2_Te_3_. (**f**) Simulated absorption spectra for devices with diverse structures and varied resonance thicknesses. (**e**,**f**) Reproduced with permission [144]. Copyright 2020, Wiley-VCH GmbH (Weinheim, Germany).

Owing to their atomic-scale dimensions, low-dimensional materials exhibit weak light absorption. By manipulating the light field, artificial optical structures can enhance localized optical fields, thereby facilitating strong interactions between light and matter. Combining artificial optical structures with low-dimensional materials effectively mitigates the inherent light absorption limitations in low-dimensional materials. Due to the nanoscale dimensions of artificial structure elements and the associated plasmonic resonance, which solely define the detection wavelength of the detector, factors such as material band gap or intrinsic absorption characteristics are inconsequential. Consequently, the integration of thermal detectors with metallic metasurfaces enables the realization of both effective absorbers and on-chip spectral filters. Dai et al. [124] leveraged the concept by employing a meticulously designed absorber structure coupled with Te nanowires, as illustrated in Figure 2a. They achieved an absorption rate exceeding 90% at 8 μm (Figure 2b), thereby enhancing the photothermal conversion efficiency. In 2022, Dai et al. [143] exploited the spatial distribution of chiral metamaterials to implement full Stokes detection (Figure 2c). By harnessing the PTE effect of the material, they achieved the conversion of absorption from different polarization lights into corresponding polarization voltage outputs at three ports, as illustrated in Figure 2d. Furthermore, the vertical optical architecture of the device contributes to enhanced light absorption. Wredh et al. [144] developed a Sb_2_Te_3_-Bi_2_Te_3_ thermocouple based on an optical resonator (Figure 2e). By modulating the size of the optical resonator, they fine-tuned the optimal detection wavelength, achieving resonant enhancement of light coupling with free electrons in the material (Figure 2f). It is important to note that adjusting the optimal detection wavelength of the device to the long-wavelength infrared (LWIR) region necessitates an increase in material thickness to augment the resonator thickness. This raises the thermal capacitance of the device, which diminishes the thermal response and speed.

**Figure 3:**
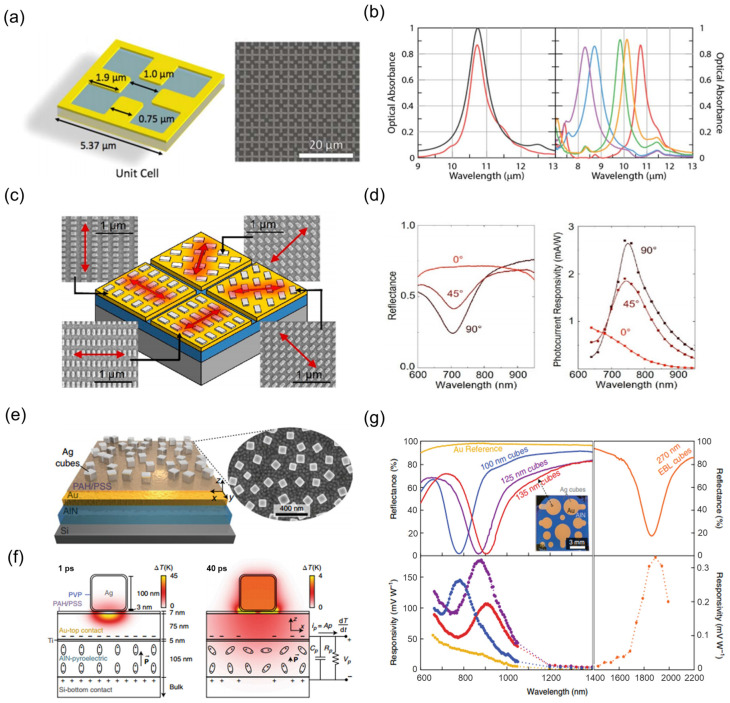
Plasmonic resonances in pyroelectric TDLMs. (**a**) Schematic diagram of the lithium niobite (LiNbO_3_) metasurface unit cell designed for a specific wavelength. The right image shows an electron micrograph of the metasurface structure at the top. (**b**) Simulated (black curve) and measured (red curve) absorption spectra of the metasurface detector. The right image displays the measured optical absorption spectra of multiple devices under various size optimizations. (**a**,**b**) Reproduced with permission [145]. Copyright 2017, Optica Publishing Group (Washington, DC, USA). (**c**) A pyroelectric detector integrating four metasurface structures optimized for different polarization angles. (**d**) Reflection spectra and response spectra of the devices at different polarization angles. (**c**,**d**) Reproduced with permission [146]. Copyright 2023, American Chemical Society (Washington, DC, USA). (**e**) Schematic diagram of a vertical structure for a photodetector based on Ag nanocube/AlN. (**f**) Thermal pulse response graph of a single metamaterial element at 1 ps and 40 ps after the excitation pulse. (**g**) Effect of Ag nanoparticle size on the reflection spectrum and response spectrum of the devices. (**e**,**g**) Reproduced with permission [147]. Copyright 2019, Springer Nature.

Besides its applications in thermoelectric materials, the design of plasmonic metasurfaces can also be integrated with pyroelectric materials, injecting novel vitality into traditional pyroelectric photodetectors. In 2017, Suen et al. [145] integrated spectrally selective metasurface unit absorbers onto thin LiNbO_3_ films (Figure 3a). As shown in Figure 3b, they achieved high narrowband absorption with a peak wavelength of 10.73 μm, full width at half maximum of 560 nm, and an absorbance of 86%. Moreover, the metallic materials of the metasurface can serve as electrodes for signal extraction, eliminating the need for an additional signal extraction component. Furthermore, a single device can be engineered to detect light at different linear polarization angles by implementing spatially segmented metasurface designs. Figure 3c illustrates the schematic structure of the design [146]. As the offset angle of the design area position varies, the peak responsivity caused by its light absorption peak also changes at specific angles, as shown in Figure 3d. In 2019, Stewart et al. [147] demonstrated a pyroelectric TDLM based on Ag nanocube plasmonic structures (Figure 3e). Under fundamental plasmon resonance, over 98% of the incident light energy is converted into localized electron density oscillations confined within the metal surface between the Ag nanocubes and the gold film. Subsequently, the localized surface plasmon decays on a femtosecond timescale, generating heat via several picosecond electron-phonon scattering processes. This heat then diffuses through the 75 nm thick gold film into the underlying AlN thermosensitive layer on a scale of tens of picoseconds. The diffusion process is illustrated by the thermal maps at different times in Figure 3f. Notably, the plasmon resonance frequency can be tuned by controlling the size of the Ag nanoparticles, enabling the adjustment of the resonant wavelength peak, as shown in Figure 3g.

#### 3.1.2. Composite Engineering

**Figure 4:**
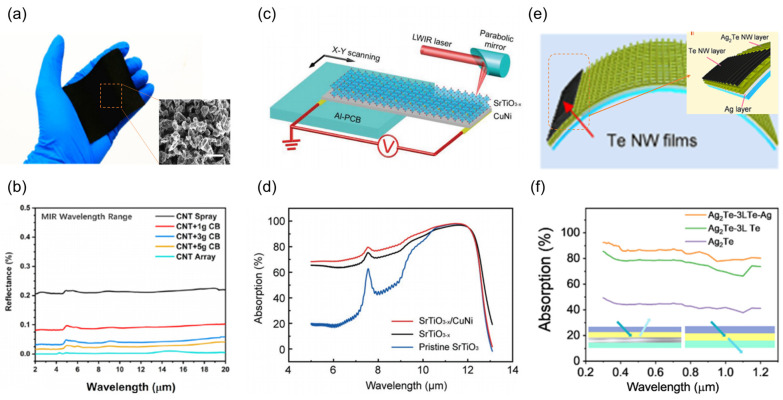
Development of high absorption materials. (**a**) Physical image of spray-coated carbon nanotube composite carbon black material. (**b**) Optimization of spectral absorption by carbon black content control. (**c**) Schematic illustration of the optoelectrical measurement for SrTiO_3-x_/CuNi composite material. (**d**) Dual optical absorption optimization strategy: SrTiO_3-x_ composition modulation and back reflection structure. (**e**) Structural diagram of the light-absorbing multilayer Ag_2_Te-Te-Ag nanofilm. (**f**) Multilayer Ag_2_Te-Te-Ag absorber spectral response. (**a**,**b**) Reproduced with permission [148]. Copyright 2021, Elsevier (Amsterdam, The Netherlands). (**c**,**d**) Reproduced with permission [149]. Copyright 2022, Wiley-VCH GmbH. (**e**,**f**) Reproduced with permission [150]. Copyright 2022, American Chemical Society.

In the field of composite engineering, researchers have significantly enhanced photothermal conversion efficiency through material compounding and structural innovation. Jin et al. [148] reported a perfect absorber coating fabricated via spray coating, comprising a multi-scale composite of carbon nanotubes (CNTs) and carbon black (CB) (Figure 4a). It has a hierarchical micro-nano pore structure made by combining 10 μm CB particles with a CNTs network that makes sub-micrometer pores. As illustrated in Figure 4b, its coating absorbs over 99.9% of light in the 400 nm–20 μm wavelength range. The Mie scattering effect of CB particles widens the absorption bandwidth. CNTs, in forming a continuous conductive network connecting the carbon black particles, optimize the surface nanostructure to enhance light harvesting. The porosity of its perfect absorber coating reduces the effective refractive index, and the CNT network facilitates carrier transport. Experimental verification of the omnidirectional absorption and self-cleaning properties of the coating demonstrates its potential in solar thermal energy collection areas. Its efficient and scalable industrial fabrication via a spray coating process fulfills practical demands.

Building upon optimized broadband light absorption, interfacial engineering has emerged as a crucial strategy for simultaneously enhancing photothermal conversion and thermoelectric transport efficiencies. Guo et al. [149] designed a SrTiO_3-x_/CuNi heterostructure TDLM (Figure 4c) that exemplifies the approach, leveraging synergistic effects of oxygen vacancy engineering and bandgap manipulation at the metal-semiconductor interface. Specifically, the perovskite SrTiO_3-x_ crystals, a prototypical oxide perovskite, exhibit a native phonon resonance absorption efficiency of 98% within the 9.5 μm wavelength (Figure 4d). Concurrently, the high electrical conductivity (5 × 10^5^ S/m) of the CuNi layer reduces the internal resistance of the device to 1.2 kΩ, effectively amplifying the Seebeck effect driven by the temperature gradient (−564 μV/K). Subsequent experimental validation demonstrated that the TDLM generates an output voltage of 13.6 mV under human body radiation (5 mW/cm^2^). The response level of the TDLM to human radiation is orders of magnitude higher than those of low-dimensional materials-based PTE detectors and even commercial thermopiles. Furthermore, the multi-layered heterogeneities effectively inhibit lateral heat diffusion, resulting in a voltage signal decay of less than 5% over 1500 s. This cascading design strategy, characterized by “photon capture-thermal localization-electric transport”, establishes a novel paradigm for energy harvesting in low-intensity radiation environments.

As device design progresses towards flexibility and miniaturization, nanoscale anisotropic control exhibits unique advantages. Wang et al. [150] fabricated a TDLM based on a Te-Ag_2_Te-Ag nanowire array, as illustrated in Figure 4e. The absorption edge can be extended to 1200 nm by utilizing the quantum confinement effect of Te nanowires (diameter 12 nm) (Figure 4f), while the preferential crystal plane alignment of Ag_2_Te results in a Seebeck coefficient of 90 μV/K. The flexible TDLM retains a responsivity of 4.1 V/W after 5000 bending cycles. Notably, surface plasmon resonance induced by the Te nanowire spacing enables polarization-sensitive detection in the ultraviolet range, surpassing the wavelength limitations of conventional grating structures. These studies collectively illuminate two key avenues for optimizing composite materials: at the microscale, by hetero-composite control of carrier transport and photothermal effects, and at the mesoscale, through multi-level structural design to enhance light harvesting and suppress thermal relaxation. Future investigations may further explore dynamically tunable composite structures to address the demand for adaptive photothermal conversion in complex operating environments.

### 3.2. Thermoelectric Enhancement Strategies

Thermoelectric conversion refers to generating a potential difference or changes in electrical conductivity due to a temperature gradient in a thermoelectric material. A substantial thermoelectric response necessitates either a high thermoelectric coefficient (Seebeck coefficient, pyroelectric coefficient, or temperature coefficient of resistance) or the deliberate creation of a significant temperature difference. Generally, the intrinsic thermoelectric coefficient of a photosensitive material within TDLMs dictates the photocurrent in TDLMs, thereby affording avenues for optimization of the thermoelectric process. Moreover, a synergistic design incorporating multiple mechanisms plays a pivotal role in amplifying the electrical signal generated through thermoelectric conversion. This discourse elucidates four strategies for optimizing thermoelectric processes, particularly emphasizing the performance enhancement of TDLMs.

#### 3.2.1. Thermoelectric Coefficient Manipulation

**Figure 5:**
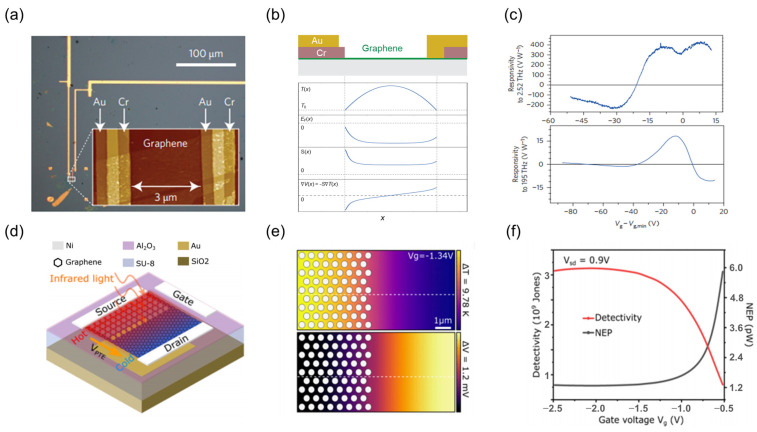
Modulation method for TDLMs Seebeck coefficient. (**a**) Optical micrograph of a graphene-based TDLM with an asymmetric electrode design. (**b**) Spatial distributions of temperature *T*(*x*), Fermi level *E*_F_(*x*), Seebeck coefficient *S*(*x*), and electric potential gradient ∇*V*(*x*) = −*S*∇*T*(*x*) along the device cross-section. (**c**) Gate voltage modulation of broadband optoelectrical response for the device. (**a**–**c**) Reproduced with permission [151]. Copyright 2014, Springer Nature. (**d**) Schematic of a TDLM based on dynamically tunable graphene. (**e**) Carrier temperature distribution map and corresponding photothermal potential distribution map on the patterned asymmetric graphene channel. (**f**) Effects of gate voltage on the specific detectivity D* and noise equivalent power *NEP* for the device. (**d**–**f**) Reproduced with permission [152]. Copyright 2024, American Chemical Society.

In thermoelectric coefficient optimization strategies, dynamic regulation of the Seebeck coefficient offers a pivotal pathway toward achieving efficient thermoelectric conversion. In 2014, Cai et al. [151] pioneered the realization of a graphene-based TDLM leveraging metal contact asymmetry, as depicted in Figure 5a. This design employs a Cr/Au heterometallic electrode configuration, generating a Fermi level gradient across the TDLM. This creates a significant Seebeck voltage via a carrier temperature gradient induced by the photothermal effect (Figure 5b). The spatial asymmetry inherent in the geometric design leads to a non-uniform distribution of the electric potential gradient, ultimately yielding a responsivity exceeding 10 V/W at room temperature, as illustrated in Figure 5c. Notably, further theoretical enhancement of the responsivity to the order of 10^5^ V/W is attainable through optimized contact resistance disparity and work function asymmetry. While metasurfaces excel in broadband spectral selectivity, heterostructures uniquely enable interfacial thermal transport modulation. This necessitates a strategic trade-off between spectral tunability and thermal efficiency when selecting device architectures. Recent advancements indicate that the synergetic combination of geometric asymmetry within a homogeneous material and dynamic electric gating enables broadband, wide-spectrum tuning with rapid response times. Guo et al. [152] furthered the development of electric gating strategies, achieving dynamic Seebeck coefficient modulation via ionic gel gating (Figure 5d). As the gate voltage shifts from −2.4 V to 0 V, the graphene Fermi level transitions from 1.0 eV to 0.2 eV, causing a redshift in the localized surface plasmon resonance wavelength from 8.5 μm to 11.2 μm. Concurrently, the gate voltage modulation synchronizes the thermal distribution and thermoelectric potential gradient of the graphene (Figure 5e). This electrostatic gating mechanism provides the device with a specific detectivity of 3.15 × 10^9^ Jones at *V*_g_ = −2 V (Figure 5f), exhibiting a rapid response time of 144 ns within the 8–12 μm spectral range.

#### 3.2.2. Thermal Management Design

TDLMs are inherently subject to three modes of heat transfer: thermal conduction, thermal convection, and thermal radiation. This perpetual energy exchange with the surroundings impedes the attainment of obvious temperature variations. Consequently, the design of TDLMs with minimized thermal dissipation is paramount to maximizing the responsivity of thermal devices.

**Figure 6:**
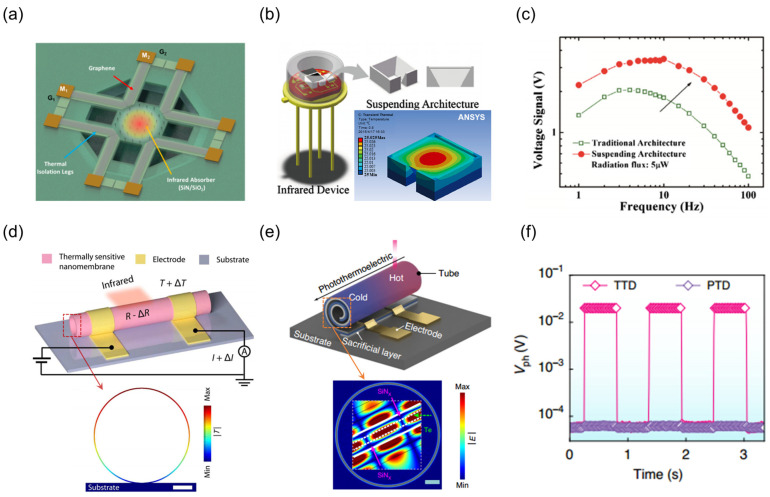
Designs of thermal regulation structure. (**a**) Schematic diagram of a suspended graphene thermopile. (**a**) Reproduced with permission [153]. Copyright 2015, American Chemical Society. (**b**) Packaging diagram of pyroelectric device based on inverted pyramid suspended structure. The illustration shows the temperature distribution of a sensitive element supported by a suspended structure. (**c**) Comparison of frequency-dependent voltage signals between suspended and planar structures. (**b**,**c**) Reproduced with permission [154]. Copyright 2016, Elsevier. (**d**) Schematic of a VO_2_ tubular bolometer based on a one-step rolling process. The inset displays the thermal distribution of the device. (**d**) Reproduced with permission [155]. Copyright 2023, The American Association for the Advancement of Science. (**e**) Schematic design of a self-rolling tubular telluride detector. The inset represents the internal electric field distribution of the multi-layered rolled structure. (**f**) Comparison of device response characteristics between planar and self-rolling structures. (**e**,**f**) Reproduced with permission [156]. Copyright 2024, Springer Nature.

To mitigate heat conduction, TDLMs can adopt the classical suspending structure design in conventional thermal devices. This is a thermal structural control strategy independent of inherent material properties. Hsu et al. [153] achieved a responsivity of 7–9 V/W and a specific detectivity of 8 × 10^8^ Jones at room temperature through the integration of graphene and Microelectromechanical Systems (MEMS) technology (Figure 6a). By utilizing the thermal isolation capabilities of silicon nitride films and the high carrier mobility of graphene, they successfully realized non-coherent imaging of blackbody targets ranging from 300 to 500 K. By optimizing the thermal resistance distribution and the thermoelectric conversion efficiency, its structural design overcomes the limitations of intrinsic material parameters, validating the universal optimization potential of MEMS fabrication in TDLMs. Furthermore, 3D printing technology offers novel avenues for fabricating suspended support structures. Xu et al. [154] employed 3D printing to construct inverted pyramid suspending structures (Figure 6b). This structure not only exhibits superior thermal isolation properties, leading to a 1.9-fold enhancement in the responsivity of the pyroelectric detector (Figure 6c), but also suppresses noise induced by ambient vibrations through edge fixation. Compared to conventional suspending structures, those fabricated via 3D printing possess greater design flexibility and lower production costs, presenting fresh perspectives for the development of high-performance infrared TDLMs.

Three-dimensional thermal structure control technology based on self-rolling nanofilms has introduced innovative design paradigms for high-sensitive TDLMs. By precisely manipulating the geometric morphology and strain distribution at the micro- and nano-scale, researchers have successfully achieved synergistic enhancements in light absorption, localized thermal management, and thermoelectric conversion efficiency. Wu et al. [155] developed a VO_2_ tubular bolometer focusing on the control of the thermal sensitivity characteristics of phase-change materials (Figure 6d). Through a one-step rolling process, the strain gradient of the VO_2_/Cr bilayer film was transformed into a thermal isolation structure, reducing the phase-transition temperature from 68 °C to 40 °C. In conjunction with the compressive strain-induced modulation of carrier mobility, a specific detectivity of 2 × 10^8^ Jones was achieved. However, the high-temperature driving characteristics of VO_2_ limit its adaptability in wide-temperature-range applications. Addressing the challenge, Huang et al. [156] constructed a telluride-based self-rolling tubular self-powered TDLM (Figure 6e) through a novel mechanism of geometrically induced energy localization. The tubular structure, facilitated by multi-layer “SiN_x_-Te-SiN_x_” heterostructure interfaces, exhibits optical resonance, thereby localizing the photonic energy within the Te layer possessing a high Seebeck coefficient (2672.72 μV/K). Compared to planar structures, the self-curling structure, through the synergy of enhanced optical absorption and low thermal conductivity design, achieves two orders of magnitude improvement in photovoltaic responsivity (Figure 6f), with a maximum photovoltaic responsivity of 252.13 V/W. Compared to suspended structures, self-rolling structures exhibit several advantages in thermal management. Firstly, thermal isolation efficiency is significantly enhanced. The three-dimensional tubular geometry formed by self-rolling effectively minimizes the contact area, suppressing the heat conduction path and reducing heat loss by an order of magnitude compared to suspended structures. Secondly, the photothermal conversion mechanism is optimized. The multiple reflections within the coiled wall generate a resonance cavity effect. This effect increases light absorption efficiency by 20-fold compared to suspended structures [156], which rely on external extensional absorption layers and limit photothermal conversion. Thirdly, mechanical stability is improved. The ring-shaped stress distribution of the coiled structure dissipates thermal expansion stress, preventing micropillar vibration or structural fracture, which can occur in suspended structures due to single-point support. Finally, process compatibility and integration are enhanced. The self-rolling technique enables one-step fabrication via strain gradients, reducing the critical process steps required for the complex photolithography-release process of suspended structures. This makes self-rolling structures more suitable for large-scale array integration.

**Figure 7:**
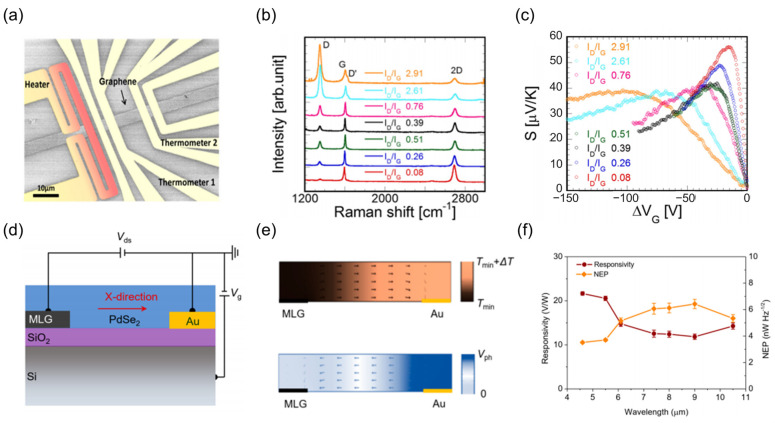
Temperature gradient control strategies. (**a**) Photograph of a graphene thermometer fabricated via defect engineering. The influence of successive increases in oxygen plasma treatment cycles on the Raman spectroscopy (**b**) and Seebeck coefficient (**c**) of graphene. The red curve depicts the untreated control group. (**a**–**c**) Reproduced with permission [157]. Copyright 2017, AIP Publishing (Melville, NY, USA). (**d**) Schematic illustration of a PdSe_2_ TDLM based on planar asymmetry thermal conductivity design. (**e**) 2D visualization of the planar temperature and photo-potential distribution in the asymmetric structure device. (**f**) MWIR to LWIR spectral detection performance of the device. (**d**–**f**) Reproduced with permission [158]. Copyright 2022, American Chemical Society.

Beyond thermal conductivity reduction for performance enhancement, temperature gradient design within the thermal transport pathway has emerged as an important strategy for further augmenting thermoelectric responses. In 2017, Anno et al. [157] employed oxygen plasma treatment to introduce controllable defects into graphene, elucidating the quantitative relationship between defect type and thermal conductivity (Figure 7a). Raman spectroscopy revealed that as the defect density shifted from *I_D_*/*I_G_* = 0.08 to 2.91, sp^3^ defects progressively transformed into vacancy-type defects (Figure 7b). This transformation resulted in a decline in lattice thermal conductivity that exceeded 40% and substantially exacerbated the localized temperature gradient. Utilizing Ioffe’s semi-classical approximation, it was observed that phonon scattering dominated the thermoelectric power at low defect densities, reaching 56 μV/K. However, charge impurity scattering reduced the maximum thermoelectric power to 38 μV/K in high-density regions (Figure 7c). This indicates that defect engineering can be used to modulate the temperature gradient distribution directionally. Building upon the foundation, Dai et al. [158] recently advanced asymmetric thermal conductivity design in Figure 7d, constructing a heterogeneous contact using Au (180 W·m^−1^·K^−1^) and multi-layer graphene (3000 W·m^−1^·K^−1^). According to Figure 7e, Micro-Raman thermography confirmed the generation of a 9.78 K temperature gradient across the device channel under 10.5 μm global illumination. As depicted in Figure 7f, the asymmetric heat dissipation design facilitated a PdSe_2_ TDLM to attain a high responsivity of 13 V/W within the 4.6–10.5 μm wavelength range. This is three times better than traditional symmetrical contact devices and keeps a low noise equivalent power of 7 nW·Hz^−1/2^.

#### 3.2.3. Intrinsic Material Properties Enhancement

**Figure 8:**
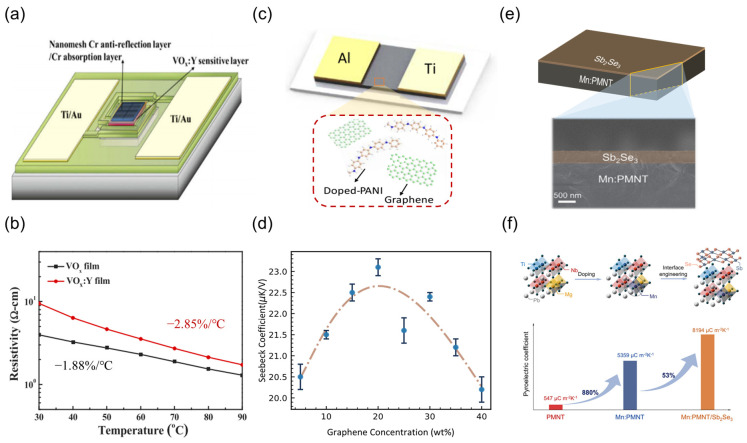
Material properties enhancement strategies. (**a**) Schematic illustration of a TDLM based on VO_x_:Y thin films. (**b**) Resistance measurements of VO_x_ and VO_x_:Y materials at various temperatures. (**a**,**b**) Reproduced with permission [159]. Copyright 2020, Optica Publishing Group. (**c**) Schematic illustration of a TDLM based on doped PANI/graphene composite. (**d**) Seebeck coefficient of the devices with varying graphene concentrations. (**c**,**d**) Reproduced with permission [160]. Copyright 2022, American Chemical Society. (**e**) Three-dimensional schematic and cross-sectional scanning electron micrograph of Mn: PMNT/Sb_2_Se_3_. (**f**) Enhancement of pyroelectric performance via doping and interfacial engineering. (**e**,**f**) Reproduced with permission [161]. Copyright 2024, Elsevier.

Enhancements in the intrinsic thermoelectric properties of materials are essential for boosting device performance, in addition to optimizing thermal transport pathways. By modulating the electronic band structure and polarization characteristics through chemical doping and interfacial engineering, the *TCR*, the pyroelectric coefficient, and the Seebeck coefficient can be augmented. Yeh et al. [159] synthesized Y-doped VO_x_ (VO_x_:Y) thin films via radio-frequency magnetron sputtering, revealing that the introduction of Y^3+^ ions promoted the transformation of V_2_O_5_ into the VO_2_ phase (Figure 8a). XPS and XRD analyses demonstrated that the Y-O bond energy is lower than the V-O bond energy, leading to oxygen vacancy reconstruction, which enhanced the *TCR* from −1.88%·K^−1^ to −2.85%·K^−1^ (Figure 8b). In conjunction with a nanogrid anti-reflective layer that reduced infrared reflectance to 18.59%, the device voltage responsivity reached 931.89 kV/W, resulting in a detectivity enhancement to 2.20 × 10^8^ Jones. Similarly, Xie et al. [160] fabricated doped PANI/graphene composite organic semiconductor materials, as illustrated in Figure 8c. Through π-π stacking, the carrier mobility of the composite material was augmented, achieving a Seebeck coefficient of 21.8 μV/K when the graphene content was optimized to 30 wt% (Figure 8d). Consequently, the maximum responsivity of the device was elevated to 2.5 V/W. Further, Guo et al. [161] epitaxially grew Sb_2_Se_3_ semiconductor layers on the surface of Mn-doped PMNT single crystals (Figure 8e), forming an interfacial band bending that generated an internal electric field. Interface lattice mismatch induced a polarization symmetry breaking, causing the pyroelectric coefficient to jump from 547 μC·m^−2^·K^−1^ to 8194 μC·m^−2^·K^−1^, as shown in Figure 8f. The synergistic effect of intrinsic polarization fields and interfacial thermoelectric effects resulted in an output power density of 41.92 mW·cm^−2^ for the device, representing a fourteen-fold increase compared to the undoped system.

#### 3.2.4. Synergistic Mechanism Engineering

**Figure 9:**
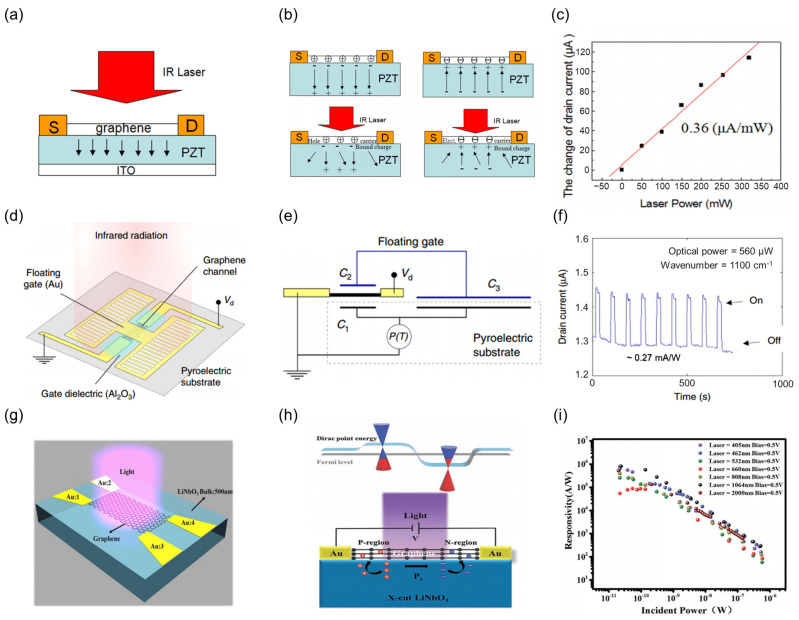
Multi-mechanism enhanced thermoelectric strategies. (**a**) Schematic diagram of a lead zirconate titanate (PZT)/graphene TDLM. (**b**) Infrared response mechanism of the device. (**c**) Current response as a function of incident light power. (**a**–**c**) Reproduced with permission [162]. Copyright 2012, AIP Publishing. (**d**) Schematic diagram of a graphene pyroelectric bolometer. (**e**) Equivalent circuit diagram of the device operational mechanism. (**f**) Infrared periodic response IT curve of the device. (**d**–**f**) Reproduced with permission [141]. Copyright 2017, Springer Nature. (**g**) Schematic diagram of an xcut-LiNbO_3_/graphene TDLM. (**h**) Band structure diagram of the homojunction PN junction of the device. (**i**) Dependence of the device photoresponsivity on incident light power density over various laser wavelengths. (**g**–**i**) Reproduced with permission [163]. Copyright 2021, Wiley-VCH GmbH.

In thermoelectric optimization strategies, the synergistic interplay of multiple physical effects can also enhance the responsivity and response time of the device. Hsieh et al. [162] first combines graphene with PZT to fabricate a PE transistor, as depicted in Figure 9a. This device leverages the synergistic coupling of the pyroelectric effect of the PZT with the photogating effect of the graphene. The thermally induced polarization changes in the PZT directly modulate the carrier concentration in graphene, resulting in an amplified thermal response current (Figure 9b). At a wavelength of 1064 nm, a responsivity of 0.36 μA/W was achieved, representing a five-order magnitude improvement over conventional SiO_2_-based devices (Figure 9c). To enhance infrared sensitivity further, Sassi et al. [141] designed a floating-gate graphene pyroelectric bolometer on an x-cut LiNbO_3_ substrate, as illustrated in Figure 9d. This design confines the charge of the pyroelectric material within the graphene channel via a floating-gate structure (Figure 9e), realizing an equivalent *TCR* of 900%·K^−1^ and a current responsivity of 0.27 mA/W (Figure 9f). To address the demands for high-speed, broadband detection, Guan et al. [163] further developed a p-n composite structure based on x-cut LiNbO_3_ (Figure 9g). Through laser-induced localized polarization modulation, both p-type and n-type doping are simultaneously achieved within the graphene (Figure 9h), creating an internal electric field that accelerates carrier separation. As shown in Figure 9i, the device exhibits a responsivity of 10^6^ A/W (1064 nm @ 24 pW) across a broad spectral range from 405 to 2000 nm. It also responds three orders of magnitude faster than conventional z-cut substrate devices, with a rise/fall time of 23 ms/23 ms.

## 4. Representative Application of TDLMs

### 4.1. Optical Communication

TDLMs exhibit promising potential for applications in high-speed optical communication due to their rapid response, high sensitivity, and self-powered capabilities [164,165,166]. TDLMs exploit the PTE to achieve efficient broadband optical-to-electrical signal conversion. Notably, the suppression of dark current at zero bias and the ultrafast charge carrier dynamics of TDLMs offer a compelling technological pathway for next-generation high-speed, low-power optical communication systems.

Optical communication systems impose stringent requirements on detector response speed and bandwidth. Conventional semiconductor detectors face limitations due to carrier mobility and recombination mechanisms, hindering their ability to surpass the 100 GHz response threshold. Graphene-based TDLMs, with their ultra-high carrier mobility (>10^5^ cm^2^·V^−1^·s^−1^) and femtosecond-scale thermal carrier relaxation times, emerge as promising candidates for breaking through the bandwidth limitations of traditional photodetectors.

**Figure 10:**
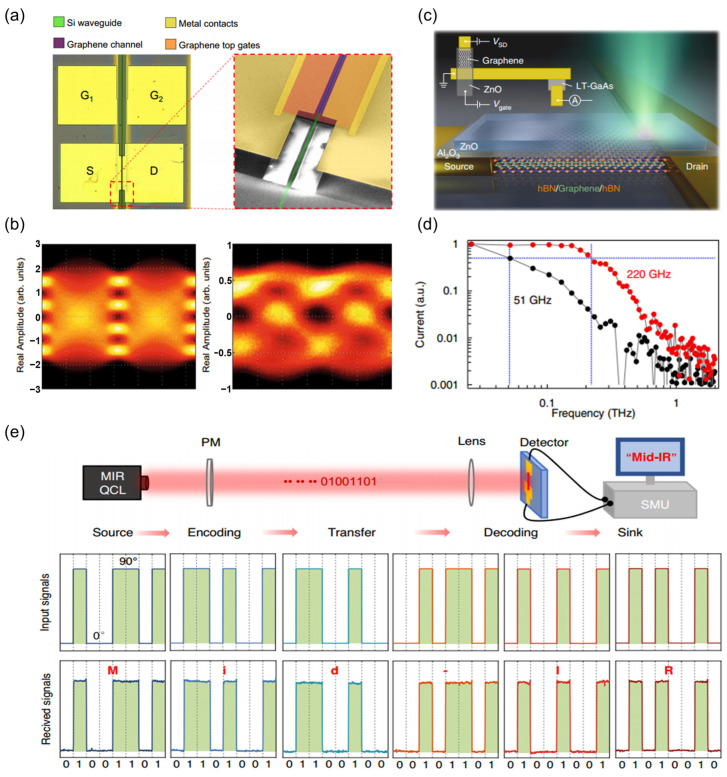
Optical communication applications of TDLMs. (**a**) Schematic diagram of graphene detectors with integrated silicon waveguide. (**b**) Eye diagrams obtained for a 60 GBaud 4-level-Pulse-Amplitude-Modulation (PAM4) and 105 GBaud Non-Return-to-Zero (NRZ) On-Off-Keying (OOK) signals. (**a**,**b**) Reproduced with permission [167]. Copyright 2021, Springer Nature. (**c**) Schematic diagram of graphene detector with on-chip ultrafast electrical readout. (**d**) Normalized fourier transform spectra of device currents. The red and black lines represent different devices of the same size. (**c**,**d**) Reproduced with permission [168]. Copyright 2022, Springer Nature. (**e**) Schematic diagram of an experimental setup of a polarization-encoding communication system. The lower graph depicts the input and device receiving end signal response plots. (**e**) Reproduced with permission [124]. Copyright 2023, Springer Nature.

Marconi et al. [167] fabricated waveguide-integrated graphene detectors via chemical vapor deposition (Figure 10a) and achieved frequency responses exceeding 65 GHz under zero bias conditions. They successfully demonstrated the direct detection of 60 Gbit/s PAM4 and 105 Gbit/s NRZ optical signals, as illustrated in Figure 10b. Their innovation stemmed from optimizing the photothermal gradient distribution and impedance matching design. This made the voltage amplification much more effective, making sure output signal integrity meets practical communication standards. This design effectively circumvents the gain-bandwidth product limitations of conventional transimpedance amplifiers at high bandwidths, offering a novel approach for ultra-high-speed optical receivers. Further investigations demonstrated that the intrinsic response time of graphene is dominated by the carrier thermalization process. Yoshioka et al. [168] utilized ultrashort pump-probe techniques coupled with on-chip terahertz electrical readout (Figure 10c) to unveil the femtosecond-scale intrinsic dynamics of graphene PTE current: photoexcited carriers form a non-equilibrium distribution during thermalization (<100 fs) [169,170,171,172], subsequently relax through phonon scattering (~4 ps) [106,169,173,174,175], and ultimately realize instantaneous current output via the Shockley–Ramo effect. The experimentally measured bandwidth of the graphene detector reached 220 GHz (Figure 10d), with the response time tunable in the sub-picosecond to picosecond range through Fermi-level engineering.

In optical communication, polarization multiplexing techniques effectively enhance channel capacity, but traditional solutions rely on complex optical components, hindering on-chip integration. Dai et al. [124] developed a highly polarization-sensitive (polarization ratio = 2.5 × 10^4^) LWIR TDLM based on one-dimensional tellurium nanoribbons and plasmonic metamaterials. The TDLM efficiently converts the photothermal gradient into an electrical signal through the synergistic effect of the high Seebeck coefficient (413 μV/K) of Te nanoribbons and the polarization-selective absorption of finite-sized plasmon resonators. At a wavelength of 8 μm, the device exhibits a responsivity of 410 V/W and a polarization angle sensitivity of 7.10 V·W^−1^·degree^−1^, representing an order of magnitude improvement over existing technologies. Moreover, a three-port device design enabled full Stokes parameter analysis of linear polarization states. Experimentally, the TDLM successfully decoded ASCII (American Standard Code for Information Interchange) polarization-modulated signals (Figure 10e), validating its practical applicability in free-space optical communication.

### 4.2. Polarization Encryption Imaging

**Figure 11:**
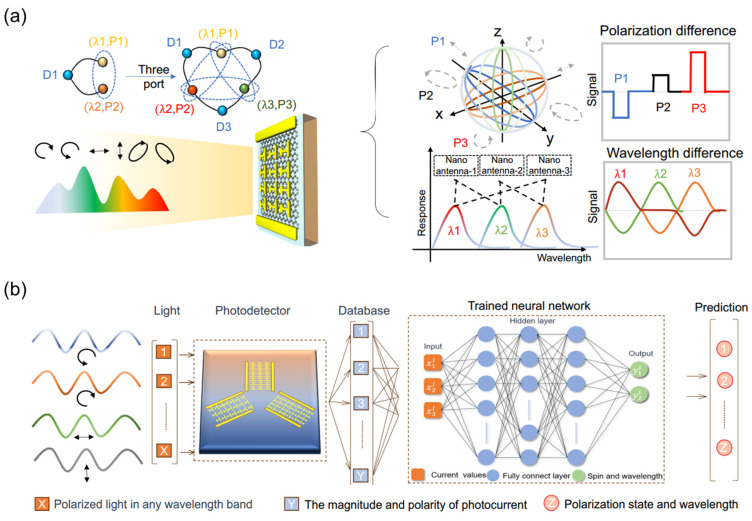
Intelligent multidimensional spectral sensing method. (**a**) Schematic diagram of methods for simultaneous perception and processing of light polarization and wavelength. (**b**) Schematic conceptualizing the machine learning approach for acquiring and identifying wavelength and polarization using a three-port system. (**a**,**b**) Reproduced with permission [176]. Copyright 2023, Springer Nature.

In the realm of polarization-based information encryption imaging, the synergistic design of metasurfaces and low-dimensional materials offers new avenues for secure communications. For instance, TDLMs co-designed with graphene and metasurfaces can decipher both polarization state and wavelength information of incident light via the polarization-sensitive PTE effect, as illustrated in Figure 11a. Jiang et al. [176] adopted a multi-port metasurface architecture coupled with machine learning algorithms (Figure 11b) to encode wavelengths and polarizations together over a wide range of 1–8 μm, predicting wavelengths with a 0.5 μm accuracy. This multidimensional signal decoupling mechanism encodes encrypted information onto polarization states, which can be subsequently decoded using customized algorithms, significantly enhancing the secrecy and anti-jamming capabilities of information transmission.

**Figure 12:**
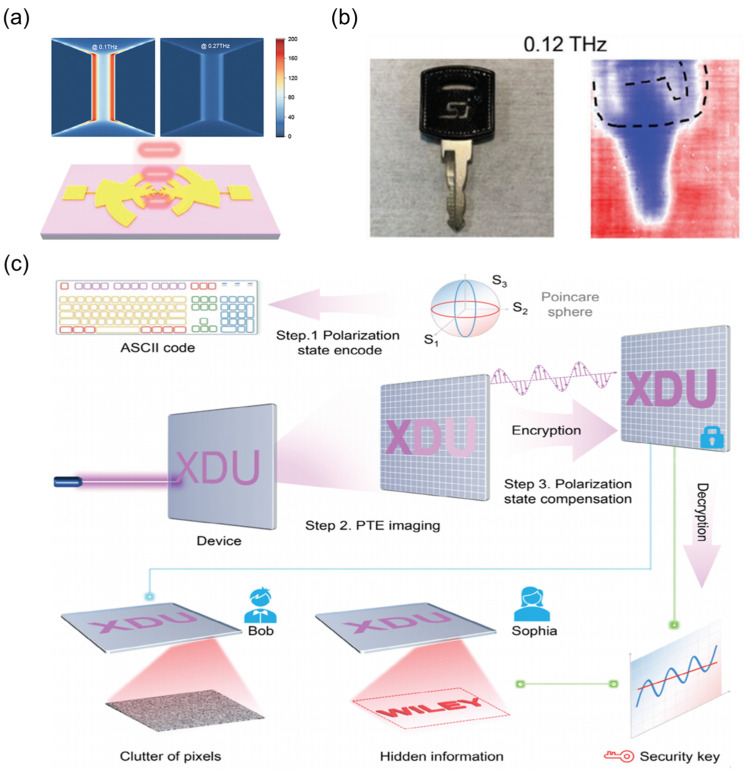
Optical encryption imaging. (**a**) Schematic diagram of a Nb_3_Se_12_I TDLM for THz detection, incorporating a planar antenna structure. (**b**) Optical image of a metallic key with a plastic top and its corresponding 0.10 THz image. (**c**) Schematic diagram of an optical encryption imaging communication system, which comprises three steps: polarization state encoding, PTE imaging, and polarization compensation. (**a**–**c**) Reproduced with permission [177]. Copyright 2024, Wiley-VCH GmbH.

Furthermore, the imaging capabilities of TDLMs across an ultra-broad spectral range (deep ultraviolet to terahertz) have spurred the development of full-band optical encryption techniques. Zhang et al. [177] fabricated a broadband TDLM based on the quasi-one-dimensional material Nb_3_Se_12_I (Figure 12a). Based on the synergistic effect of PTE and photoconductive effects, the fabricated detector exhibits a broad spectral response spanning from deep ultraviolet (254 nm) to terahertz (0.30 THz) frequencies, enabling rapid and precise broadband imaging. For example, the integration of terahertz penetration imaging with visible light polarization compensation enables the covert analysis of internal structures within metallic objects, as illustrated in Figure 12b. Through polarization state encoding, PTE imaging, and polarization compensation (Figure 12c), encrypted information can be embedded within multi-band imaging results. These technologies have applications not only in military and defense but also provide a highly compatible platform for multispectral biomedical diagnostics [178,179,180,181,182,183].

### 4.3. Flexible Sensing

Low-dimensional materials, endowed with distinctive quantum confinement effects, high carrier mobility, and mechanical flexibility, present unprecedented opportunities for developing high-performance flexible TDLMs. Among these, PTE detectors, leveraging the photothermal effect to directly convert infrared radiation into electrical signals without external biasing, exhibit several compelling advantages, including full spectral response, room-temperature operation, and self-powering capabilities. These attributes position them as particularly promising candidates for applications in flexible electronic skin [184,185,186,187,188], temperature sensing [189,190,191,192,193], and health electronics [194,195,196,197,198].

**Figure 13:**
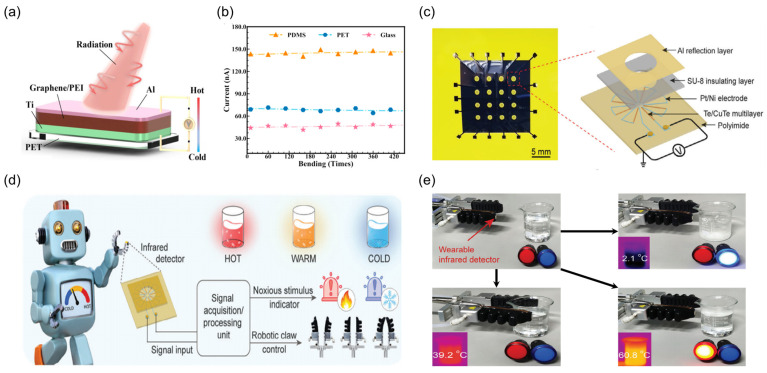
Flexible applications. (**a**) Schematic diagram of a graphene/PEI composite PTE detector. (**b**) Photocurrent of the device with different substrates under multiple bending cycles. (**a**,**b**) Reproduced with permission [199]. Copyright 2023, American Chemical Society. (**c**) Image of a flexible infrared TDLM arrays for contactless thermal compensation with spatial resolution. The inset shows a schematic diagram of the asymmetric reflective thermoelectric pile structure based on Te/CuTe multilayers. (**d**) Demonstration of the application of the wearable infrared TDLM in a robotic temperature warning function. (**e**) Physical image of a flexible mechanical gripper with a flexible infrared TDLM attached to the tip of the claw. The right side shows its response to water at low (2.1 °C), warm (39.2 °C), and high (60.8 °C) temperatures. (**c**–**e**) Reproduced with permission [200]. Copyright 2024, Wiley-VCH GmbH.

In the realm of flexible sensing, Xie et al. [199] employed a spray coating technique to fabricate a graphene/PEI TDLM (Figure 13a), showcasing its high photothermal conversion capabilities on a flexible substrate. By meticulously controlling the solution viscosity (PEI concentration of 800 mg/mL) and graphene (10 wt%), a uniform coating with a thickness of 4.78 ± 0.5 μm was achieved, leading to a 40% reduction in resistance compared to the drop-casting method. Notably, the PEI matrix effectively lowered the thermal conductivity (≈0.12 W·m^−1^·K^−1^) while simultaneously enhancing π-π interactions between graphene molecules, resulting in an average Seebeck coefficient of −31.5 μV/K. Under irradiation from a 973 K blackbody source (peak wavelength 2.98 μm), the detector exhibited a responsivity of 2.7 V/W and a detectivity of 6.05 × 10^7^ Jones, surpassing most polymer-based detectors. Furthermore, the TDLM demonstrated remarkable bending durability, exhibiting a response decay of less than 10% after 30 days of operation at 90% humidity and retaining its performance integrity even after 400 bending cycles (Figure 13b). Guo et al. [200] presented a 4 × 4 asymmetric reflective TDLM arrays based on Te/CuTe multilayered heterostructures (Figure 13c). By employing magnetron sputtering to alternatively deposit Te and Cu layers followed by annealing to form a CuTe interface, the carrier mobility was significantly enhanced, resulting in a conductivity enhancement to 1.6 × 10^3^ S/m compared to pristine Te thin films, while retaining a high Seebeck coefficient of 312 μV/K. The multilayered structure further amplified the temperature gradient response through the incorporation of a low thermal conductivity polyimide (PI) film (≈0.12 W·m^−1^·K^−1^). Moreover, fine-tuning the multilayered film thickness (periodicity n = 7) to match the absorption peak of the PI substrate (C-O-C stretching mode, corresponding to 1195 cm^−1^) induced destructive interference of the reflected light waves, leading to an absorption enhancement to 87% in the LWIR band (8–14 μm). This flexible sensor can be intelligently integrated into robotic systems. Specifically, the Te/CuTe TDLM arrays was integrated into a robotic gripper, successfully demonstrating a robot-based thermal warning system (Figure 13d). When the device was affixed to the front end of the robotic arm and approached different temperature water sources (Figure 13e), the system could accurately identify thermal hazards in real-time based on the voltage signals generated from the thermal radiation differences and trigger corresponding avoidance maneuvers. Furthermore, its output signal can span a dynamic temperature range of –50~110 °C, with a response time as low as 154 ms, fulfilling the robotic requirements for rapid thermal feedback. In conjunction with infrared thermography, the systems can also resolve the spatial contours of radiation through masking imaging, extending applications to human health monitoring and industrial non-destructive testing.

## 5. Perspective and Outlook

In recent years, research in the field of TDLMs has made significant strides. The unique material properties of these materials offer novel approaches to circumvent the limitations of conventional thermal imaging techniques. While interfacial engineering and metasurface integration have demonstrably broadened spectral response ranges and enabled detection capabilities at room temperature, there are still areas that need further optimization. These areas include functional integrity, environmental robustness, and system-level integration for practical applications.

At the fundamental physical level, the mechanisms underlying the performance enhancement of TDLMs through multi-field coupling remain an active area of exploration. While existing research has preliminarily unveiled the potential of phenomena such as localized thermal field enhancement via plasmonic and thermal transport facilitated by topological material edge states, the synergistic mechanisms between these effects and the thermoelectric properties of low-dimensional materials require deeper investigation. Future advancements may arise from combining multi-scale simulations and high spatiotemporal-resolution characterization techniques. This synergistic approach could elucidate the energy conversion dynamics under extreme conditions, such as high-frequency alternating thermal fluxes and multi-physics coupling, thereby guiding the design of low-noise, high-responsivity devices. Notably, the quantum confinement effects inherent to low-dimensional materials and the intricate interplay with metasurfaces hold promise for broadband detection spanning the infrared to terahertz spectral ranges. Nevertheless, theoretical models and experimental validations are currently in the conceptual stage and necessitate further exploration.

From the perspectives of material systems and device architectures, existing challenges predominantly center on balancing thermal transport efficiency and sensitivity. Although interfacial engineering in heterojunctions has demonstrably improved light-to-heat conversion capabilities, precise control over thermal relaxation processes remains a hurdle. For instance, incorporating dynamically responsive thermal materials, such as phase-change modulation layers or flexible thermal-conductive films, may offer novel solutions for adaptive thermal flow management. Yet, their long-term stability and environmental compatibility require rigorous validation. For example, the rapid oxidation of black phosphorus under ambient conditions remains a key limitation. Recent advances in encapsulation (e.g., hBN passivation) show promise but require further validation for industrial adoption. To address these challenges, cross-disciplinary collaboration must bridge materials innovation with industrial manufacturing standards, particularly in standardizing synthesis protocols (e.g., CVD growth uniformity) and scalable integration techniques for heterogeneous material systems.

In practical applications, thermal detection technology aims to evolve from “passive perception” to “active decision-making”. Currently, research emphasis is progressively shifting from optimizing individual device performance to constructing multi-functional sensing networks. Conventional thermal imaging systems are constrained by latency and power consumption in their posterior algorithms. Conversely, neuromorphic computing architectures, embodied in sensing-compute-in-memory chips, enable on-site processing of thermal signals through “compute-in-memory” design. For instance, integrating pulse-driven TDLM arrays with memristor crossbar networks on a single chip can emulate the parallel processing mechanisms of biological visual systems, directly outputting thermal feature encodings of target objects. Such systems hold significant value for applications like autonomous driving and industrial inspections. However, a key challenge lies in realizing high-fidelity transmission and efficient fusion of multi-modal thermal signals. Furthermore, coupling TDLMs with energy-harvesting modules can lead to self-powered intelligent sensor networks. These networks can sustain power generation through ambient temperature gradients, enabling real-time monitoring and feedback regulation of remote devices. This technology offers comprehensive, low-power solutions for smart city development, remote healthcare, and other scenarios requiring round-the-clock operation.

In conclusion, the development of TDLMs exhibiting both high sensitivity and intelligent thermal management, coupled with multi-functional detection capable of wide-spectrum optoelectronic response, represents a crucial direction for future research. Hence, alongside investigating the intrinsic merits of novel low-dimensional materials, further exploration of synergistic mechanisms in heterojunctions, concurrently integrating micro-nano engineering strategies, is essential for enhancing TDLMs performance and expanding its application domain.

## References

[1] Wu W.F., Ma H., Cai X., Han B., Li Y., Xu K., Lin H.T., Zhang F., Chen Z.Y., Zhang Z.Y. (2023). High-Speed Carbon Nanotube Photodetectors for 2 μm Communications. ACS Nano.

[2] Zhu Y., Chen H., Han R., Qin H., Yao Z., Liu H., Ma Y., Wan X., Li G., Chen Y. (2024). High-speed flexible near-infrared organic photodiode for optical communication. Natl. Sci. Rev..

[3] Fu J.T., Nie C.B., Sun F.Y., Jiang H., Li Y.J., Li G.L., Wei X.Z. (2023). Photo-Driven Semimetal-Semiconductor Field-Effect Transistors. Adv. Opt. Mater..

[4] Fu J.T., Ji L., Wu Z.C., Li G.L., Nie C.B., Xiong W., Wang F., Sun F.Y., Zhou Y.C., Zang Z.G. (2024). An all-in-one optoelectronic logic device with self-distinguishable dual-band photoresponse. Device.

[5] Yang C., Feng S.L., Tang L.L., Shen J., Wei X.Z., Shi H.F. (2021). Electrochemical Epitaxial Grown PbS Nanorods Array on Graphene Film for High-Performance Photodetector. Adv. Mater. Interfaces.

[6] Wu D., Guo C.G., Zeng L.H., Ren X.Y., Shi Z.F., Wen L., Chen Q., Zhang M., Li X.J., Shan C.X. (2023). Phase-controlled van der Waals growth of wafer-scale 2D MoTe_2_ layers for integrated high-sensitivity broadband infrared photodetection. Light Sci. Appl..

[7] Zeng L.H., Han W., Ren X.Y., Li X., Wu D., Liu S.J., Wang H., Lau S.P., Tsang Y.H., Shan C.X. (2023). Uncooled Mid-Infrared Sensing Enabled by Chip-Integrated Low-Temperature-Grown 2D PdTe_2_ Dirac Semimetal. Nano Lett..

[8] Wu D., Mo Z.H., Li X., Ren X.Y., Shi Z.F., Li X.J., Zhang L., Yu X.C., Peng H.X., Zeng L.H. (2024). Integrated mid-infrared sensing and ultrashort lasers based on wafer-level Td-WTe_2_ Weyl semimetal. Appl. Phys. Rev..

[9] Li Z.A., Si G.S., Ning Z.Q., Liu J.X., Fang Y.H., Si B.B., Cheng Z., Yang C.P. (2022). Highly Sensitive Sphere-Tube Coupled Photoacoustic Cell Suitable for Detection of a Variety of Trace Gases: NO_2_ as an Example. Sensors.

[10] Wang F.K., Zhu S., Chen W.D., Han J.Y., Duan R.H., Wang C.W., Dai M.J., Sun F.Y., Jin Y.H., Wang Q.J. (2024). Multidimensional detection enabled by twisted black arsenic-phosphorus homojunctions. Nat. Nanotechnol..

[11] Zhang S.K., Jiao H.X., Chen Y., Yin R.T., Huang X.N., Zhao Q.R., Tan C., Huang S.Y., Yan H.G., Lin T. (2024). Multi-dimensional optical information acquisition based on a misaligned unipolar barrier photodetector. Nat. Commun..

[12] Zeng L.H., Wu D., Jie J.S., Ren X.Y., Hu X., Lau S.P., Chai Y., Tsang Y.H. (2020). Van der Waals Epitaxial Growth of Mosaic-Like 2D Platinum Ditelluride Layers for Room-Temperature Mid-Infrared Photodetection up to 10.6 μm. Adv. Mater..

[13] Wu P.S., Ye L., Tong L., Wang P., Wang Y., Wang H.L., Ge H.N., Wang Z., Gu Y., Zhang K. (2022). Van der Waals two-color infrared photodetector. Light Sci. Appl..

[14] Zhou Y., Qiu X., Wan Z.A., Long Z.H., Poddar S., Zhang Q.P., Ding Y.C., Chan C.L.J., Zhang D.Q., Zhou K.M. (2022). Halide-exchanged perovskite photodetectors for wearable visible-blind ultraviolet monitoring. Nano Energy.

[15] Lee K., Cho I., Kang M.G., Jeong J., Choi M., Woo K.Y., Yoon K., Cho Y., Park I. (2023). Ultra-Low-Power E-Nose System Based on Multi-Micro-LED-Integrated, Nanostructured Gas Sensors and Deep Learning. Acs Nano.

[16] Wei S.Y., Li Z., Murugappan K., Li Z.Y., Zhang F.L., Saraswathyvilasam A.G., Lysevych M., Tan H.H., Jagadish C., Tricoli A. (2023). A Self-Powered Portable Nanowire Array Gas Sensor for Dynamic NO_2_ Monitoring at Room Temperature. Adv. Mater..

[17] Yu J., Zheng J., Chen H.Y., Tian N., Li L., Qu Y.M., Huang Y.T., Luo Y.X., Tan W.Z. (2021). Near-infrared photodetectors based on CH_3_NH_3_PbI_3_ perovskite single crystals for bioimaging applications. J. Mater. Chem. C.

[18] He Y.M., Hu Y.X., Peng M., Fu L.C., Gao E.R., Liu Z.Y., Dong C., Li S., Ge C.Y., Yuan C. (2024). One-Dimensional Crystal-Structure Te-Se Alloy for Flexible Shortwave Infrared Photodetector and Imaging. Nano Lett..

[19] Hu W.D., Ye Z.H., Liao L., Chen H.L., Chen L., Ding R.J., He L., Chen X.S., Lu W. (2014). 128 x 128 long-wavelength/mid-wavelength two-color HgCdTe infrared focal plane array detector with ultralow spectral cross talk. Opt. Lett..

[20] Peng S.L., Zhang C.Y., Wei Y.C., Ouyang Y., Han J.Y., Li C.Y., Dong M.D., Wang J. (2025). High performance self-powered PbSe/WSe_2_ p-n heterojunction photodetector for image sensing. J. Mater. Sci. Technol..

[21] Wang T., Zheng D.M., Vegso K., Mrkyvkova N., Siffalovic P., Yuan X.C., Somekh M.G., Coolen L., Pauporte T., Fu F. (2023). Flexible array of high performance and stable formamidinium-based low-n 2D halide perovskite photodetectors for optical imaging. Nano Energy.

[22] Zhuang R.Z., Cai S.H., Mei Z.X., Liang H.L., Zhao N.J., Mu H.R., Yu W.Z., Jiang Y., Yuan J., Lau S.P. (2023). Solution-grown BiI/BiI_3_ van der Waals heterostructures for sensitive X-ray detection. Nat. Commun..

[23] Han J.Y., Deng W.J., Hu F.C., Han S., Wang Z., Fu Z.Y., Zhou H.X., Yu H., Gou J., Wang J. (2025). 2D Materials-Based Photodetectors with Bi-Directional Responses in Enabling Intelligent Optical Sensing. Adv. Funct. Mater..

[24] Zeng L.H., Wu D., Lin S.H., Xie C., Yuan H.Y., Lu W., Lau S.P., Chai Y., Luo L.B., Li Z.J. (2019). Controlled Synthesis of 2D Palladium Diselenide for Sensitive Photodetector Applications. Adv. Funct. Mater..

[25] Yang M., Wang J., Han J.Y., Ling J.W., Ji C.H., Kong X., Liu X.C., Huang Z.H., Gou J., Liu Z.J. (2018). Enhanced Performance of Wideband Room Temperature Photodetector Based on Cd_3_As_2_ Thin Film/Pentacene Heterojunction. ACS Photonics.

[26] Jiang H., Wei J.X., Sun F.Y., Nie C.B., Fu J.T., Shi H.F., Sun J.X., Wei X.Z., Qiu C.W. (2022). Enhanced Photogating Effect in Graphene Photodetectors via Potential Fluctuation Engineering. ACS Nano.

[27] Han J.Y., Wang J., Yang M., Kong X., Chen X.Q., Huang Z.H., Guo H., Gou J., Tao S.L., Liu Z.J. (2018). Graphene/Organic Semiconductor Heterojunction Phototransistors with Broadband and Bi-directional Photoresponse. Adv. Mater..

[28] Fu J.T., Guo Z.M., Nie C.B., Sun F.Y., Li G.L., Feng S.L., Wei X.Z. (2024). Schottky infrared detectors with optically tunable barriers beyond the internal photoemission limit. Innovation.

[29] Jiang H., Wang M., Fu J.T., Li Z.C., Shaikh M.S., Li Y.J., Nie C.B., Sun F.Y., Tang L.L., Yang J. (2022). Ultrahigh Photogain Short-Wave Infrared Detectors Enabled by Integrating Graphene and Hyperdoped Silicon. ACS Nano.

[30] Jiang H., Nie C.B., Fu J.T., Tang L.L., Shen J., Sun F.Y., Sun J.X., Zhu M., Feng S.L., Liu Y. (2020). Ultrasensitive and fast photoresponse in graphene/silicon-on-insulator hybrid structure by manipulating the photogating effect. Nanophotonics.

[31] Fu J.T., Yang C., Nie C.B., Sun F.Y., Li G.L., Feng S.L., Wei X.Z. (2023). Vertical Photodetectors Based on In Situ Aligned Single-crystalline PbS Nanocuboids Sandwiched between Graphene Electrodes. Adv. Opt. Mater..

[32] Vashishtha P., Dash A., Walia S., Gupta G. (2025). Self-bias Mo–Sb–Ga multilayer photodetector encompassing ultra-broad spectral response from UV–C to IR–B. Opt. Laser Technol..

[33] Vashishtha P., Dash A., Prajapat P., Goswami P., Walia S., Gupta G. (2023). Self-Powered Broadband Photodetection of MoS_2_/Sb_2_Se_3_ Heterostructure. ACS Appl. Opt. Mater..

[34] Zhang H., Zhao K.Y., Cui S.Y., Yang J., Zhou D.H., Tang L.L., Shen J., Feng S.L., Zhang W.G., Fu Y.Q. (2018). Anomalous temperature coefficient of resistance in graphene nanowalls/polymer films and applications in infrared photodetectors. Nanophotonics.

[35] He R.H., Chen Z.F., Lai H.J., Zhang T.K., Wen J.X., Chen H.J., Xie F.Y., Yue S., Liu P.Y., Chen J. (2019). Van der Waals Transition-Metal Oxide for Vis-MIR Broadband Photodetection via Intercalation Strategy. ACS Appl. Mater. Interfaces.

[36] Li G., Yin S.Q., Tan C.Y., Chen L.J., Yu M.X., Li L., Yan F. (2021). Fast Photothermoelectric Response in CVD-Grown PdSe_2_ Photodetectors with In-Plane Anisotropy. Adv. Funct. Mater..

[37] Sun X.L., Sheng Y., Gao X., Liu Y., Ren F., Tan Y., Yang Z.X., Jia Y.C., Chen F. (2022). Self-Powered Lithium Niobate Thin-Film Photodetectors. Small.

[38] Lu Y.Y., Liu L.Y., Gao R.Q., Xiong Y., Sun P.Q., Wu Z.H., Wu K., Yu T., Zhang K., Zhang C. (2024). Ultrafast near-infrared pyroelectric detector based on inhomogeneous plasmonic metasurface. Light Sci. Appl..

[39] Zhu B.X., Zhu C.Y., Qin J.K., He W., Yue L.Q., Huang P.Y., Li D., Sun R.Y., Ye S., Du Y. (2024). Two-dimensional SnP_2_Se_6_ with gate-tunable Seebeck coefficient for telecommunication band photothermoelectric detection. InfoMat.

[40] Niu T.Y., Morais N., Qiu B.Q., Nagai N., Zhang Y., Arakawa Y., Hirakawa K. (2021). GaAs-based microelectromechanical terahertz bolometers fabricated on high-resistivity Si substrates using wafer bonding technique. Appl. Phys. Lett..

[41] An P.P., Kovalyuk V.V., Gladush Y.G., Golikov A.D., Semenov A.V., Komrakova S.A., Ozhegov R.V., Mkrtchyan A.A., Krasnikov D.V., Nasibulin A.G. (2024). High-speed optical-waveguide integrated single-walled carbon nanotube bolometer. Appl. Phys. Lett..

[42] Gunyho A.M., Kundu S., Ma J., Liu W., Niemela S., Catto G., Vadimov V., Vesterinen V., Singh P., Chen Q.M. (2024). Single-shot readout of a superconducting qubit using a thermal detector. Nat. Electron..

[43] Goto M., Yamada Y., Shimura A., Suzuki T., Degawa N., Yamane T., Aoki S., Urabe J., Hara S., Nomura H. (2021). Uncooled sub-GHz spin bolometer driven by auto-oscillation. Nat. Commun..

[44] Tong J.Y., Muthee M., Chen S.Y., Yngvesson S.K., Yan J. (2015). Antenna Enhanced Graphene THz Emitter and Detector. Nano Lett..

[45] Tong J.Y., Conte M.C., Goldstein T., Yngvesson S.K., Bardin J.C., Yan J. (2018). Asymmetric Two-Terminal Graphene Detector for Broadband Radiofrequency Heterodyne- and Self-Mixing. Nano Lett..

[46] Erikson K.J., He X.W., Talin A.A., Mills B., Hauge R.H., Iguchi T., Fujimura N., Kawano Y., Kono J., Léonard F. (2015). Figure of Merit for Carbon Nanotube Photothermoelectric Detectors. Acs Nano.

[47] Zhao D., Fabiano S., Berggren M., Crispin X. (2017). Ionic thermoelectric gating organic transistors. Nat. Commun..

[48] Liu S.L., Ding Y.J., Rong W.Z., Xu Y., Li Y.W., Onwudiwe D.C., Bae B.S., Ertugrul M., Zhu Y., Wu Z. (2024). Working Voltage Switching the Photo-/Thermo-Electric Effect for Distinct Ultraviolet and Infrared Signal Detection. Acs Nano.

[49] Wu D., Guo J.W., Du J., Xia C.X., Zeng L.H., Tian Y.Z., Shi Z.F., Tian Y.T., Li X.J., Tsang Y.H. (2019). Highly Polarization-Sensitive, Broadband, Self-Powered Photodetector Based on Graphene/PdSe_2_/Germanium Heterojunction. ACS Nano.

[50] Hu W.D., Chen X.S., Ye Z.H., Lu W. (2011). A hybrid surface passivation on HgCdTe long wave infrared detector with in-situ CdTe deposition and high-density hydrogen plasma modification. Appl. Phys. Lett..

[51] Gao Z., Jiang S.Y., Huang P.H., Chen Y., Qi Q.Y., Zhao H.Q., Wei X.Z., Shi H.F., Zhao X., Xiao Z.Y. (2024). Integrating self-assembled supramolecular organic frameworks with inorganic semiconductors for infrared photodetection. Aggregate.

[52] Yang J., Tang L.L., Luo W., Shen J., Zhou D.H., Feng S.L., Wei X.Z., Shi H.F. (2019). Light Trapping in Conformal Graphene/Silicon Nanoholes for High-Performance Photodetectors. ACS Appl. Mater. Interfaces.

[53] Zeng L.H., Chen Q.M., Zhang Z.X., Wu D., Yuan H.Y., Li Y.Y., Qarony W., Lau S.P., Luo L.B., Tsang Y.H. (2019). Multilayered PdSe_2_/Perovskite Schottky Junction for Fast, Self-Powered, Polarization-Sensitive, Broadband Photodetectors, and Image Sensor Application. Adv. Sci..

[54] Yang M., Wang J., Zhao Y.F., He L., Ji C.H., Liu X.C., Zhou H.X., Wu Z.M., Wang X.R., Jiang Y.D. (2019). Three-Dimensional Topological Insulator Bi_2_Te_3_/Organic Thin Film Heterojunction Photodetector with Fast and Wideband Response from 450 to 3500 Nanometers. ACS Nano.

[55] Vashishtha P., Abidi I.H., Giridhar S.P., Verma A.K., Prajapat P., Bhoriya A., Murdoch B.J., Tollerud J.O., Xu C.L., Davis J.A. (2024). CVD-Grown Monolayer MoS_2_ and GaN Thin Film Heterostructure for a Self-Powered and Bidirectional Photodetector with an Extended Active Spectrum. ACS Appl. Mater. Interfaces.

[56] Vashishtha P., Prajapat P., Kumar K., Kumar M., Walia S., Gupta G. (2023). Multiband spectral response inspired by ultra-high responsive thermally stable and self-powered Sb_2_Se_3_/GaN heterojunction based photodetector. Surf. Interfaces.

[57] Su J.T., Li C.Y., Xiao J.H., Kong J.C., Hu P.Y., Lu C.G., Zhu L. (2023). Boosting infrared absorption through surface plasmon resonance enhanced HgCdTe microcavity. J. Appl. Phys..

[58] Ma H., Wu J.H., Wang Y.P., Zhong C.Y., Ye Y.T., Wei M.L., Yu R., Du Y.Q., Tang B., Sun C. (2022). Enhanced Light-Tellurium Interaction through Evanescent Wave Coupling for High Speed Mid-Infrared Photodetection. Adv. Opt. Mater..

[59] Zhu L.Q., Wu T.X., Wang Z.H., Wang X., Li X., Zhou S.M., Gan Z.K., Lin C., Chen B.L. (2024). Low Dark Current HgCdTe Long Wavelength Infrared Photodiodes With Bandgap Gradient Multi-Layer Heterojunction. IEEE Electron Device Lett..

[60] Jones A.H., March S.D., Bank S.R., Campbell J.C. (2020). Low-noise high-temperature AlInAsSb/GaSb avalanche photodiodes for 2-μm applications. Nat. Photonics.

[61] Babicheva V.E., Lock E., Kim H. (2024). Narrow-bandgap titanium sesquioxide with resonant metasurfaces for enhanced infrared absorption. Appl. Phys. Lett..

[62] Chen D.K., March S.D., Jones A.H., Shen Y., Dadey A.A., Sun K.Y., McArthur J.A., Skipper A.M., Xue X.J., Guo B.T. (2023). Photon-trapping-enhanced avalanche photodiodes for mid-infrared applications. Nat. Photonics.

[63] Xiao L., Nie C.B., Jiang Y.Y., Fu J.T., Zhu P., Li N., Wang G.W., Shi H.F., Wei X.Z., Sun T. (2024). Ultralow-Noise MoS_2_/Type II Superlattice Mixed-Dimensional van der Waals Barrier Long-Wave Infrared Detector. ACS Appl. Mater. Interfaces.

[64] Cao G.Q., Li T., Tang H.J., Shao X.M., Li X., Gong H.M. (2014). Performance of Extended Wavelength InGaAs/InAsP SWIR Detector.

[65] Camargo E., Ueno K., Kawakami Y., Moriyasu Y., Nagase K., Sato M., Endo H., Ishibashi K., Kuze N. (2008). Miniaturized InSb photovoltaic infrared sensor operating at room temperature. Opt. Eng..

[66] McCullough P.R., Regan M., Bergeron L., Lindsay K. (2008). Quantum efficiency and quantum yield of an HgCdTe infrared sensor array. Publ. Astron. Soc. Pac..

[67] Tonouchi M. (2007). Cutting-edge terahertz technology. Nat. Photonics.

[68] Chan W.L., Deibel J., Mittleman D.M. (2007). Imaging with terahertz radiation. Rep. Prog. Phys..

[69] Zhang R., Yang Z.J., Liu L.W., Lin J., Wen S.F., Meng Y., Yin Y., Lan C.Y., Li C., Liu Y. (2023). Highly Sensitive Broadband Bolometric Photodetectors based on 2D PdSe_2_ Thin Film. Adv. Opt. Mater..

[70] Huang Y.T., Zheng T., Wu J.G. (2024). KNN composite ceramics with superior pyroelectric performance for self-powered thermal detector. Nano Energy.

[71] Hou X.H., Liu Y., Bai S.Y., Yu S.J., Huang H., Yang K., Li C., Peng Z.X., Zhao X.L., Zhou X.Z. (2024). Pyroelectric Photoconductive Diode for Highly Sensitive and Fast DUV Detection. Adv. Mater..

[72] Ahmadi R., Abnavi A., Ghanbari H., Mohandes H., Mohammadzadeh M.R., De Silva T., Hasani A., Fawzy M., Kabir F., Adachi M.M. (2022). Self-powered, broadband, and polarization-sensitive pyroelectric-photoelectric photodetector based on silicon-water heterojunction. Nano Energy.

[73] Li L.J., O’Farrell E.C.T., Loh K.P., Eda G., Ozyilmaz B., Neto A.H.C. (2016). Controlling many-body states by the electric-field effect in a two-dimensional material. Nature.

[74] Gong C., Li L., Li Z.L., Ji H.W., Stern A., Xia Y., Cao T., Bao W., Wang C.Z., Wang Y. (2017). Discovery of intrinsic ferromagnetism in two-dimensional van der Waals crystals. Nature.

[75] Akinwande D., Huyghebaert C., Wang C., Serna M.I., Goossens S., Li L., Wong H.S.P., Koppens F.H.L. (2019). Graphene and two-dimensional materials for silicon technology. Nature.

[76] Burch K.S., Mandrus D., Park J. (2018). Magnetism in two-dimensional van der Waals materials. Nature.

[77] Wu W.Z., Wang L., Li Y.L., Zhang F., Lin L., Niu S.M., Chenet D., Zhang X., Hao Y.F., Heinz T.F. (2014). Piezoelectricity of single-atomic-layer MoS_2_ for energy conversion and piezotronics. Nature.

[78] Meyer J.C., Geim A.K., Katsnelson M.I., Novoselov K.S., Booth T.J., Roth S. (2007). The structure of suspended graphene sheets. Nature.

[79] Kishida H., Matsuzaki H., Okamoto H., Manabe T., Yamashita M., Taguchi Y., Tokura Y. (2000). Gigantic optical nonlinearity in one-dimensional Mott-Hubbard insulators. Nature.

[80] Philp E., Sloan J., Kirkland A.I., Meyer R.R., Friedrichs S., Hutchison J.L., Green M.L.H. (2003). An encapsulated helical one-dimensional cobalt iodide nanostructure. Nat. Mater..

[81] Jansen C., Wietzke S., Astley V., Mittleman D.M., Koch M. (2010). Mechanically flexible polymeric compound one-dimensional photonic crystals for terahertz frequencies. Appl. Phys. Lett..

[82] Yang T.Y., Wan Q., Yan D.Y., Zhu Z., Wang Z.W., Peng C., Huang Y.B., Yu R., Hu J., Mao Z.Q. (2020). Directional massless Dirac fermions in a layered van der Waals material with one-dimensional long-range order. Nat. Mater..

[83] Kargar F., Barani Z., Sesing N.R., Mai T.T., Debnath T., Zhang H.R., Liu Y.H., Zhu Y.B., Ghosh S., Biacchi A.J. (2022). Elemental excitations in MoI_3_ one-dimensional van der Waals nanowires. Appl. Phys. Lett..

[84] Lee B.J., Fu C.J., Zhang Z.M. (2005). Coherent thermal emission from one-dimensional photonic crystals. Appl. Phys. Lett..

[85] Besombes L., Kheng K., Marsal L., Mariette H. (2001). Acoustic phonon broadening mechanism in single quantum dot emission. Phys. Rev. B.

[86] de Arquer F.G., Talapin D.V., Klimov V.I., Arakawa Y., Bayer M., Sargent E.H. (2021). Semiconductor quantum dots: Technological progress and future challenges. Science.

[87] Grundmann M., Stier O., Bimberg D. (1995). InAs/GaAs pyramidal quantum dots—Strain distribution, optical phonons, and electronic—Structure. Phys. Rev. B.

[88] Peng X.G., Manna L., Yang W.D., Wickham J., Scher E., Kadavanich A., Alivisatos A.P. (2000). Shape control of CdSe nanocrystals. Nature.

[89] Reed M.A., Randall J.N., Aggarwal R.J., Matyi R.J., Moore T.M., Wetsel A.E. (1988). Observation of discrete electronic states in a zero-dimensional semiconductor nanostructure. Phys. Rev. Lett..

[90] Srivastava A., Sidler M., Allain A.V., Lembke D.S., Kis A., Imamoglu A. (2015). Optically active quantum dots in monolayer WSe_2_. Nat. Nanotechnol..

[91] Alivisatos A.P. (1996). Semiconductor Clusters, Nanocrystals, and Quantum Dots. Science.

[92] Novoselov K.S., Mishchenko A., Carvalho A., Castro Neto A.H. (2016). 2D materials and van der Waals heterostructures. Science.

[93] Yang J., Tang L.L., Luo W., Feng S.L., Leng C.Q., Shi H.F., Wei X.Z. (2021). Interface Engineering of a Silicon/Graphene Heterojunction Photodetector via a Diamond-Like Carbon Interlayer. ACS Appl. Mater. Interfaces.

[94] Fu J.T., Jiang H., Nie C.B., Sun F.Y., Tang L.L., Li Y.J., Li Z.C., Xiong W., Yang J., Li X. (2023). Polarity-Tunable Field Effect Phototransistors. Nano Lett..

[95] Wu D., Guo J.W., Wang C.Q., Ren X.Y., Chen Y.S., Lin P., Zeng L.H., Shi Z.F., Li X.J., Shan C.X. (2021). Ultrabroadband and High-Detectivity Photodetector Based on WS_2_/Ge Heterojunction through Defect Engineering and Interface Passivation. ACS Nano.

[96] Gao Z., Leng C.Q., Zhao H.Q., Wei X.Z., Shi H.F., Xiao Z.Y. (2024). The Electrical Behaviors of Grain Boundaries in Polycrystalline Optoelectronic Materials. Adv. Mater..

[97] Zhao J.H., Tang L.B., Xiang J.Z., Ji R.B., Yuan J., Zhao J., Yu R.Y., Tai Y.J., Song L.Y. (2014). Chlorine doped graphene quantum dots: Preparation, properties, and photovoltaic detectors. Appl. Phys. Lett..

[98] Kavrik M.S., Hachtel J.A., Ko W., Qian C., Abelson A., Unlu E.B., Kashyap H., Li A., Idrobo J.C., Law M. (2022). Emergence of distinct electronic states in epitaxially-fused PbSe quantum dot superlattices. Nat. Commun..

[99] Balandin A.A. (2011). Thermal properties of graphene and nanostructured carbon materials. Nat. Mater..

[100] Boukai A.I., Bunimovich Y., Tahir-Kheli J., Yu J.K., Goddard W.A., Heath J.R. (2008). Silicon nanowires as efficient thermoelectric materials. Nature.

[101] Fong K.C., Schwab K.C. (2012). Ultrasensitive and Wide-Bandwidth Thermal Measurements of Graphene at Low Temperatures. Phys. Rev. X.

[102] Fong K.C., Wollman E.E., Ravi H., Chen W., Clerk A.A., Shaw M.D., Leduc H.G., Schwab K.C. (2013). Measurement of the Electronic Thermal Conductance Channels and Heat Capacity of Graphene at Low Temperature. Phys. Rev. X.

[103] Novoselov K.S., Geim A.K., Morozov S.V., Jiang D., Zhang Y., Dubonos S.V., Grigorieva I.V., Firsov A.A. (2004). Electric Field Effect in Atomically Thin Carbon Films. Science.

[104] Aamir M.A., Moore J.N., Lu X.B., Seifert P., Englund D., Fong K.C., Efetov D.K. (2021). Ultrasensitive Calorimetric Measurements of the Electronic Heat Capacity of Graphene. Nano Lett..

[105] Crossno J., Shi J.K., Wang K., Liu X.M., Harzheim A., Lucas A., Sachdev S., Kim P., Taniguchi T., Watanabe K. (2016). Observation of the Dirac fluid and the breakdown of the Wiedemann-Franz law in graphene. Science.

[106] Gabor N.M., Song J.C.W., Ma Q., Nair N.L., Taychatanapat T., Watanabe K., Taniguchi T., Levitov L.S., Jarillo-Herrero P. (2011). Hot Carrier–Assisted Intrinsic Photoresponse in Graphene. Science.

[107] Efetov D.K., Shiue R.J., Gao Y.D., Skinner B., Walsh E.D., Choi H., Zheng J.B., Tan C., Grosso G., Peng C. (2018). Fast thermal relaxation in cavity-coupled graphene bolometers with a Johnson noise read-out. Nat. Nanotechnol..

[108] Fei Z., Palomaki T., Wu S., Zhao W., Cai X., Sun B., Nguyen P., Finney J., Xu X., Cobden D.H. (2017). Edge conduction in monolayer WTe_2_. Nat. Phys..

[109] Geim A.K., Grigorieva I.V. (2013). Van der Waals heterostructures. Nature.

[110] Deng D.H., Novoselov K.S., Fu Q., Zheng N.F., Tian Z.Q., Bao X.H. (2016). Catalysis with two-dimensional materials and their heterostructures. Nat. Nanotechnol..

[111] Ferrari A.C., Bonaccorso F., Fal’ko V., Novoselov K.S., Roche S., Boggild P., Borini S., Koppens F.H.L., Palermo V., Pugno N. (2015). Science and technology roadmap for graphene, related two-dimensional crystals, and hybrid systems. Nanoscale.

[112] Liu Y., Weiss N.O., Duan X.D., Cheng H.C., Huang Y., Duan X.F. (2016). Van der Waals heterostructures and devices. Nat. Rev. Mater..

[113] Liu C.S., Chen H.W., Hou X., Zhang H., Han J., Jiang Y.G., Zeng X.Y., Zhang D.W., Zhou P. (2019). Small footprint transistor architecture for photoswitching logic and in situ memory. Nat. Nanotechnol..

[114] Liu C.S., Yan X., Song X.F., Ding S.J., Zhang D.W., Zhou P. (2018). A semi-floating gate memory based on van der Waals heterostructures for quasi-non-volatile applications. Nat. Nanotechnol..

[115] Li D., Chen M.Y., Sun Z.Z., Yu P., Liu Z., Ajayan P.M., Zhang Z.X. (2017). Two-dimensional non-volatile programmable p-n junctions. Nat. Nanotechnol..

[116] Li X., Wu G.L., Zhang L.N., Huang D.P., Li Y.Q., Zhang R.Q., Li M., Zhu L., Guo J., Huang T.L. (2022). Single-crystal two-dimensional material epitaxy on tailored non-single-crystal substrates. Nat. Commun..

[117] Chen Y.F., Wang Y., Wang Z., Gu Y., Ye Y., Chai X.L., Ye J.F., Chen Y., Xie R.Z., Zhou Y. (2021). Unipolar barrier photodetectors based on van der Waals heterostructures. Nat. Electron..

[118] Bao W., Jing L., Jing V., Lee Y., Liu G., Tran D., Standley B., Aykol M., Cronin S.B., Smirnov D. (2011). Stacking-dependent band gap and quantum transport in trilayer graphene. Nat. Phys..

[119] Craciun M.F., Russo S., Yamamoto M., Oostinga J.B., Morpurgo A.F., Tarucha S. (2009). Trilayer graphene is a semimetal with a gate-tunable band overlap. Nat. Nanotechnol..

[120] Lui C.H., Li Z.Q., Mak K.F., Cappelluti E., Heinz T.F. (2011). Observation of an electrically tunable band gap in trilayer graphene. Nat. Phys..

[121] Bistritzer R., MacDonald A.H. (2011). Moire bands in twisted double-layer graphene. Proc. Natl. Acad. Sci. USA.

[122] Cao Y., Fatemi V., Demir A., Fang S.A., Tomarken S.L., Luo J.Y., Sanchez-Yamagishi J.D., Watanabe K., Taniguchi T., Kaxiras E. (2018). Correlated insulator behaviour at half-filling in magic-angle graphene superlattices. Nature.

[123] Kim S.E., Mujid F., Rai A., Eriksson F., Suh J., Poddar P., Ray A., Park C., Fransson E., Zhong Y. (2021). Extremely anisotropic van der Waals thermal conductors. Nature.

[124] Dai M.J., Wang C.W., Qiang B., Jin Y.H., Ye M., Wang F.K., Sun F.Y., Zhang X.R., Luo Y., Wang Q.J. (2023). Long-wave infrared photothermoelectric detectors with ultrahigh polarization sensitivity. Nat. Commun..

[125] Koepfli S.M., Baumann M., Gadola R., Nashashibi S., Koyaz Y., Rieben D., Güngör A.C., Doderer M., Keller K., Fedoryshyn Y. (2024). Controlling photothermoelectric directional photocurrents in graphene with over 400 GHz bandwidth. Nat. Commun..

[126] Wei J.X., Chen Y., Li Y., Li W., Xie J.S., Lee C.K., Novoselov K.S., Qiu C.W. (2023). Geometric filterless photodetectors for mid-infrared spin light. Nat. Photonics.

[127] Yao Y., Kats M.A., Genevet P., Yu N.F., Song Y., Kong J., Capasso F. (2013). Broad Electrical Tuning of Graphene-Loaded Plasmonic Antennas. Nano Lett..

[128] Zhou Y., Wang C., Xu D.H., Fan R.H., Zhang K., Peng R.W., Hu Q., Wang M. (2014). Tuning the dispersion relation of a plasmonic waveguide via graphene contact. Europhys. Lett..

[129] Zhang J., Wei X.Z., Rukhlenko I.D., Chen H.T., Zhu W.R. (2020). Electrically Tunable Metasurface with Independent Frequency and Amplitude Modulations. ACS Photonics.

[130] Xiao L., Fu J.T., Zhu P., Li N., Jiang Y.Y., Shi H.F., Wei X.Z., Xiong W., Wang G.W., Sun T. (2024). Pixel-integrated Mie metasurface long-wave multispectral type II superlattice detector. Appl. Phys. Lett..

[131] Ge S.F., Li C.K., Zhang Z.M., Zhang C.L., Zhang Y.D., Qiu J., Wang Q.S., Liu J.K., Jia S., Feng J. (2015). Dynamical Evolution of Anisotropic Response in Black Phosphorus under Ultrafast Photoexcitation. Nano Lett..

[132] Guo P.J., Schaller R.D., Ketterson J.B., Chang R.P.H. (2016). Ultrafast switching of tunable infrared plasmons in indium tin oxide nanorod arrays with large absolute amplitude. Nat. Photonics.

[133] Driscoll T., Palit S., Qazilbash M.M., Brehm M., Keilmann F., Chae B.G., Yun S.J., Kim H.T., Cho S.Y., Jokerst N.M. (2008). Dynamic tuning of an infrared hybrid-metamaterial resonance using vanadium dioxide. Appl. Phys. Lett..

[134] Wu S.Q., Chen Y., Wang X.D., Jiao H.X., Zhao Q.R., Huang X.N., Tai X.C., Zhou Y., Chen H., Wang X.J. (2022). Ultra-sensitive polarization-resolved black phosphorus homojunction photodetector defined by ferroelectric domains. Nat. Commun..

[135] Biswas S., Grajower M.Y., Watanabe K., Taniguchi T., Atwater H.A. (2021). Broadband electro-optic polarization conversion with atomically thin black phosphorus. Science.

[136] Lee C., Wei X., Kysar J.W., Hone J. (2008). Measurement of the Elastic Properties and Intrinsic Strength of Monolayer Graphene. Science.

[137] Cutler M., Mott N.F. (1969). Observation of Anderson Localization in an Electron Gas. Phys. Rev..

[138] Wang F., Zhang T., Xie R.Z., Wang Z., Hu W.D. (2023). How to characterize figures of merit of two-dimensional photodetectors. Nat. Commun..

[139] Fu J.T., Nie C.B., Sun F.Y., Li G.L., Wei X.Z. (2023). Photodetectors Based on Graphene-Semiconductor Hybrid Structures: Recent Progress and Future Outlook. Adv Devices Instrum..

[140] Lu X.W., Sun L., Jiang P., Bao X.H. (2019). Progress of Photodetectors Based on the Photothermoelectric Effect. Adv. Mater..

[141] Sassi U., Parret R., Nanot S., Bruna M., Borini S., De Fazio D., Zhao Z., Lidorikis E., Koppens F.H., Ferrari A.C. (2017). Graphene-based mid-infrared room-temperature pyroelectric bolometers with ultrahigh temperature coefficient of resistance. Nat. Commun..

[142] Rogalski A. (2010). Infrared Detectors.

[143] Dai M.J., Wang C.W., Qiang B., Wang F.K., Ye M., Han S., Luo Y., Wang Q.J. (2022). On-chip mid-infrared photothermoelectric detectors for full-Stokes detection. Nat. Commun..

[144] Wredh S., Dai M.J., Hamada K., Rahman M.A., Adanan N.Q., Zamiri G., Wu Q.Y.S., Zhai W.H., Mun N.W.L., Dong Z.G. (2024). Sb_2_Te_3_-Bi_2_Te_3_ Direct Photo-Thermoelectric Mid-Infrared Detection. Adv. Opt. Mater..

[145] Suen J.Y., Fan K.B., Montoya J., Bingham C., Stenger V., Sriram S., Padilla W.J. (2017). Multifunctional metamaterial pyroelectric infrared detectors. Optica.

[146] Wilson N.C., Shin E., Bangle R.E., Nikodemski S.B., Vella J.H., Mikkelsen M.H. (2023). Ultrathin Pyroelectric Photodetector with Integrated Polarization-Sensing Metasurface. Nano Lett..

[147] Stewart J.W., Vella J.H., Li W., Fan S.H., Mikkelsen M.H. (2020). Ultrafast pyroelectric photodetection with on-chip spectral filters. Nat. Mater..

[148] Jin Y.H., Zhang T.F., Zhao J., Zhao Y.X., Liu C., Song J., Hao X.P., Wang J.P., Jiang K.L., Fan S.S. (2021). Spray coating of a perfect absorber based on carbon nanotube multiscale composites. Carbon.

[149] Guo X.H., Lu X.W., Jiang P., Bao X.H. (2022). SrTiO_3_/CuNi-Heterostructure-Based Thermopile for Sensitive Human Radiation Detection and Noncontact Human–Machine Interaction. Adv. Mater..

[150] Wang R., He Z., Wang J.L., Liu J.Y., Liu J.W., Yu S.H. (2022). Manipulating Nanowire Structures for an Enhanced Broad-Band Flexible Photothermoelectric Photodetector. Nano Lett..

[151] Cai X., Sushkov A.B., Suess R.J., Jadidi M.M., Jenkins G.S., Nyakiti L.O., Myers-Ward R.L., Li S., Yan J., Gaskill D.K. (2014). Sensitive room-temperature terahertz detection via the photothermoelectric effect in graphene. Nat. Nanotechnol..

[152] Guo T.Y., Chandra S., Dasgupta A., Shabbir M.W., Biswas A., Chanda D. (2024). Spectrally Tunable Ultrafast Long Wave Infrared Detection at Room Temperature. Nano Lett..

[153] Hsu A.L., Herring P.K., Gabor N.M., Ha S., Shin Y.C., Song Y., Chin M., Dubey M., Chandrakasan A.P., Kong J. (2015). Graphene-Based Thermopile for Thermal Imaging Applications. Nano Lett..

[154] Xu Q., Zhao X.Y., Li X.B., Deng H., Yan H., Yang L.R., Di W.N., Luo H.S., Neumann N. (2016). 3D-Printing of inverted pyramid suspending architecture for pyroelectric infrared detectors with inhibited microphonic effect. Infrared Phys. Technol..

[155] Wu B.M., Zhang Z.Y., Chen B.X., Zheng Z., You C.Y., Liu C., Li X., Wang J.L., Wang Y.Q., Song E.M. (2023). One-step rolling fabrication of VO_2_ tubular bolometers with polarization-sensitive and omnidirectional detection. Sci. Adv..

[156] Huang J.Y., You C.Y., Wu B.M., Wang Y.Q., Zhang Z.Y., Zhang X.Y., Liu C., Huang N.G., Zheng Z., Wu T.Q. (2024). Enhanced photothermoelectric conversion in self-rolled tellurium photodetector with geometry-induced energy localization. Light Sci. Appl..

[157] Anno Y., Takeuchi M., Matsuoka M., Takei K., Akita S., Arie T. (2017). Effect of defect-induced carrier scattering on the thermoelectric power of graphene. Appl. Phys. Lett..

[158] Dai M.J., Wang C.W., Ye M., Zhu S., Han S., Sun F.Y., Chen W.D., Jin Y.H., Chua Y.D., Wang Q.J. (2022). High-Performance, Polarization-Sensitive, Long-Wave Infrared Photodetection via Photothermoelectric Effect with Asymmetric van der Waals Contacts. ACS Nano.

[159] Yeh T.H., Tsai C.K., Chu S.Y., Lee H.Y., Lee C.T. (2020). Performance improvement of Y-doped VO_x_ microbolometers with nanomesh antireflection layer. Opt. Express.

[160] Xie Z.M., Wang J.Q., Yeow J.T.W. (2022). Doped Polyaniline/Graphene Composites for Photothermoelectric Detectors. ACS Appl. Nano Mater..

[161] Guo C., Xu L., Wang D.X., Huang H.B., Qian W.Q., Dan H.Y., Bowen C.R., Yang Y. (2024). Giant pyroelectricity via doping and interface engineering. Joule.

[162] Hsieh C.Y., Chen Y.T., Tan W.J., Chen Y.F., Shih W.Y., Shih W.H. (2012). Graphene-lead zirconate titanate optothermal field effect transistors. Appl. Phys. Lett..

[163] Guan H.Y., Hong J.Y., Wang X.L., Ming J.Y., Zhang Z.L., Liang A.J., Han X.Y., Dong J.L., Qiu W.T., Chen Z. (2021). Broadband, high-sensitivity graphene photodetector based on ferroelectric polarization of lithium niobate. Adv. Opt. Mater..

[164] Zhu Y., Wang B.Y., Deng C.C., Wang Y.F., Wang X.F. (2021). Photothermal-pyroelectric-plasmonic coupling for high performance and tunable band-selective photodetector. Nano Energy.

[165] Ouyang T., Zhao X., Xun X.C., Gao F.F., Zhao B., Bi S.X., Li Q., Liao Q.L., Zhang Y. (2023). Boosting Charge Utilization in Self-Powered Photodetector for Real-Time High-Throughput Ultraviolet Communication. Adv. Sci..

[166] Ding Z.P., Su W., Luo Y.L., Ye L.P., Li W.L., Zhou Y.H., Zou J.F., Tang B., Yao H.B. (2024). Metasurface inverse designed by deep learning for quasi-entire terahertz wave absorption. Nanoscale.

[167] Marconi S., Giambra M.A., Montanaro A., Miseikis V., Soresi S., Tirelli S., Galli P., Buchali F., Templ W., Coletti C. (2021). Photo thermal effect graphene detector featuring 105 Gbit s^−1^ NRZ and 120 Gbit s^−1^ PAM4 direct detection. Nat. Commun..

[168] Yoshioka K., Wakamura T., Hashisaka M., Watanabe K., Taniguchi T., Kumada N. (2022). Ultrafast intrinsic optical-to-electrical conversion dynamics in a graphene photodetector. Nat. Photonics.

[169] Tielrooij K.J., Piatkowski L., Massicotte M., Woessner A., Ma Q., Lee Y., Myhro K.S., Lau C.N., Jarillo-Herrero P., van Hulst N.F. (2015). Generation of photovoltage in graphene on a femtosecond timescale through efficient carrier heating. Nat. Nanotechnol..

[170] George P.A., Strait J., Dawlaty J., Shivaraman S., Chandrashekhar M., Rana F., Spencer M.G. (2008). Ultrafast Optical-Pump Terahertz-Probe Spectroscopy of the Carrier Relaxation and Recombination Dynamics in Epitaxial Graphene. Nano Lett..

[171] Brida D., Tomadin A., Manzoni C., Kim Y.J., Lombardo A., Milana S., Nair R.R., Novoselov K.S., Ferrari A.C., Cerullo G. (2013). Ultrafast collinear scattering and carrier multiplication in graphene. Nat. Commun..

[172] Rohde G., Stange A., Müller A., Behrendt M., Oloff L.P., Hanff K., Albert T.J., Hein P., Rossnagel K., Bauer M. (2018). Ultrafast Formation of a Fermi-Dirac Distributed Electron Gas. Phys. Rev. Lett..

[173] Song J.C.W., Rudner M.S., Marcus C.M., Levitov L.S. (2011). Hot Carrier Transport and Photocurrent Response in Graphene. Nano Lett..

[174] Tielrooij K.J., Hesp N.C.H., Principi A., Lundeberg M.B., Pogna E.A.A., Banszerus L., Mics Z., Massicotte M., Schmidt P., Davydovskaya D. (2018). Out-of-plane heat transfer in van der Waals stacks through electron–hyperbolic phonon coupling. Nat. Nanotechnol..

[175] Bistritzer R., MacDonald A.H. (2009). Electronic Cooling in Graphene. Phys. Rev. Lett..

[176] Jiang H., Chen Y.Z., Guo W.Y., Zhang Y., Zhou R.G., Gu M.L., Zhong F., Ni Z.H., Lu J.P., Qiu C.W. (2024). Metasurface-enabled broadband multidimensional photodetectors. Nat. Commun..

[177] Zhang J.B., Hu Z., Yang Q.Y., Sun S.W., Liu F., Xu H., Wang X., Zhao Y., Zhou N., Dong G.Z. (2024). Strong Anisotropy and Giant Photothermoelectricity of 1D Alloy Nb_3_Se_12_I-Based Photodetector for Ultrabroadband Light-Detection and Encryption Imaging Application. Adv. Mater..

[178] Baker M.J., Faulds K. (2016). Fundamental developments in clinical infrared and Raman spectroscopy. Chem. Soc. Rev..

[179] Liang L.L., Wang C.W., Chen J.Y., Wang Q.J., Liu X.G. (2022). Incoherent broadband mid-infrared detection with lanthanide nanotransducers. Nat. Photonics.

[180] Li X., Wu S.E., Wu D., Zhao T.X., Lin P., Shi Z.F., Tian Y.T., Li X.J., Zeng L.H., Yu X.C. (2024). In situ construction of PtSe/Ge Schottky junction array with interface passivation for broadband infrared photodetection and imaging. InfoMat.

[181] Cao F., Chen J.D., Yu D.J., Wang S., Xu X.B., Liu J.X., Han Z.Y., Huang B., Gu Y., Choy K.L. (2020). Bionic Detectors Based on Low-Bandgap Inorganic Perovskite for Selective NIR-I Photon Detection and Imaging. Adv. Mater..

[182] Wang P., Liu S.S., Luo W.J., Fang H.H., Gong F., Guo N., Chen Z.G., Zou J., Huang Y., Zhou X.H. (2017). Arrayed Van Der Waals Broadband Detectors for Dual-Band Detection. Adv. Mater..

[183] Wang F.K., Hu F.C., Dai M.J., Zhu S., Sun F.Y., Duan R.H., Wang C.W., Han J.Y., Deng W.J., Chen W.D. (2023). A two-dimensional mid-infrared optoelectronic retina enabling simultaneous perception and encoding. Nat. Commun..

[184] Shi S., Ming Y., Wu H.B., Zhi C.W., Yang L.T., Meng S., Si Y.F., Wang D., Fei B., Hu J.L. (2024). A Bionic Skin for Health Management: Excellent Breathability, In Situ Sensing, and Big Data Analysis. Adv. Mater..

[185] Zhuang Q.N., Yao K.M., Zhang C., Song X., Zhou J.K., Zhang Y.F., Huang Q.Y., Zhou Y.Z., Yu X.G., Zheng Z.J. (2024). Permeable, three-dimensional integrated electronic skins with stretchable hybrid liquid metal solders. Nat. Electron..

[186] Nela L., Tang J.S., Cao Q., Tulevski G., Han S.J. (2018). Large-Area High-Performance Flexible Pressure Sensor with Carbon Nanotube Active Matrix for Electronic Skin. Nano Lett..

[187] Lee S., Kim J., Yun I., Bae G.Y., Kim D., Park S., Yi I.M., Moon W., Chung Y., Cho K. (2019). An ultrathin conformable vibration-responsive electronic skin for quantitative vocal recognition. Nat. Commun..

[188] Gandla S., Chae H., Kwon H.J., Won Y., Park H., Lee S., Song J., Baek S., Hong Y.D., Kim D. (2022). Ultrafast Prototyping of Large-Area Stretchable Electronic Systems by Laser Ablation Technique for Controllable Robotic Arm Operations. IEEE Trans. Ind. Electron..

[189] Lu Y.F., Zhang H.J., Zhao Y., Liu H.D., Nie Z.T., Xu F., Zhu J.X., Huang W. (2024). Robust Fiber-Shaped Flexible Temperature Sensors for Safety Monitoring with Ultrahigh Sensitivity. Adv. Mater..

[190] Kang M., Qu R.X., Sun X.W., Yan Y.D., Ma Z.J., Wang H., Yan K.F., Zhang W.F., Deng Y. (2023). Self-Powered Temperature Electronic Skin Based on Island-Bridge Structure and Bi-Te Micro-Thermoelectric Generator for Distributed Mini-Region Sensing. Adv. Mater..

[191] Wang J.W., Zheng Y.P., Cui T.Y., Huang T.Y., Liu H.D., Zhu J.X., Song L., Hu Y. (2024). Bioinspired Ultra-Robust Ionogels Constructed with Soft-Rigid Confinement Space for Multimodal Monitoring Electronics. Adv. Funct. Mater..

[192] Zhang W., Wang P.L., Huang L.Z., Guo W.Y., Zhao J.J., Ma M.G. (2023). A stretchable, environmentally tolerant, and photoactive liquid metal/MXene hydrogel for high performance temperature monitoring, human motion detection and self-powered application. Nano Energy.

[193] Seo B.S., Hwang H.Y., Kang S.G., Cha Y.S., Choi W.J. (2018). Flexible-detachable dual-output sensors of fluid temperature and dynamics based on structural design of thermoelectric materials. Nano Energy.

[194] Guo Y., Zhong M.J., Fang Z.W., Wan P.B., Yu G.H. (2019). A Wearable Transient Pressure Sensor Made with MXene Nanosheets for Sensitive Broad-Range Human-Machine Interfacing. Nano Lett..

[195] Zhu Y.Z., Haghniaz R., Hartel M.C., Guan S.H., Bahari J., Li Z.J., Baidya A., Cao K., Gao X.X., Li J.H. (2023). A Breathable, Passive-Cooling, Non-Inflammatory, and Biodegradable Aerogel Electronic Skin for Wearable Physical-Electrophysiological-Chemical Analysis. Adv. Mater..

[196] Yin F.F., Guo Y.J., Li H., Yue W.J., Zhang C.W., Chen D., Geng W., Li Y., Gao S., Shen G.Z. (2022). A waterproof and breathable Cotton/rGO/CNT composite for constructing a layer-by-layer structured multifunctional flexible sensor. Nano Res..

[197] Wu W.T., Li L.L., Li Z.X., Sun J.Z., Wang L.L. (2023). Extensible Integrated System for Real-Time Monitoring of Cardiovascular Physiological Signals and Limb Health. Adv. Mater..

[198] Sun C., Liu X.R., Yao Q.X., Jiang Q., Xia X.L., Shen Y.F., Ye X.Y., Tan H.W., Gao R.S., Zhu X.J. (2023). A Discolorable Flexible Synaptic Transistor for Wearable Health Monitoring. ACS Nano.

[199] Xie Z.M., Wang J.Q., Yeow J.W. (2023). Flexible Multi-Element Photothermoelectric Detectors Based on Spray-Coated Graphene/Polyethylenimine Composites for Nondestructive Testing. ACS Appl. Mater. Interfaces.

[200] Guo X.H., Lu X.W., Jiang P., Bao X.H. (2024). Touchless Thermosensation Enabled by Flexible Infrared Photothermoelectric Detector for Temperature Prewarning Function of Electronic Skin. Adv. Mater..

